# Supratentorial non-*RELA*, *ZFTA*-fused ependymomas: a comprehensive phenotype genotype correlation highlighting the number of zinc fingers in *ZFTA-NCOA1/2* fusions

**DOI:** 10.1186/s40478-021-01238-y

**Published:** 2021-08-13

**Authors:** Arnault Tauziède-Espariat, Aurore Siegfried, Yvan Nicaise, Thomas Kergrohen, Philipp Sievers, Alexandre Vasiljevic, Alexandre Roux, Edouard Dezamis, Chiara Benevello, Marie-Christine Machet, Sophie Michalak, Chloe Puiseux, Francisco Llamas-Gutierrez, Pierre Leblond, Franck Bourdeaut, Jacques Grill, Christelle Dufour, Léa Guerrini-Rousseau, Samuel Abbou, Volodia Dangouloff-Ros, Nathalie Boddaert, Raphaël Saffroy, Lauren Hasty, Ellen Wahler, Mélanie Pagès, Felipe Andreiuolo, Emmanuèle Lechapt, Fabrice Chrétien, Thomas Blauwblomme, Kévin Beccaria, Johan Pallud, Stéphanie Puget, Emmanuelle Uro-Coste, Pascale Varlet, the RENOCLIP-LOC, the BIOMECA (Biomarkers for Ependymomas in Children, Adolescents) consortium

**Affiliations:** 1 Department of Neuropathology, GHU Paris-Psychiatrie et Neurosciences, Sainte-Anne Hospital, 1, rue Cabanis, 75014 Paris, France; 2 Institut de Psychiatrie et Neurosciences de Paris (IPNP), UMR S1266, INSERM, IMA-BRAIN, Paris, France; 3 Université de Paris, Paris, France; 4 Department of Pathology, Toulouse University Hospital, Toulouse, France; 5 INSERM U1037, Cancer Research Center of Toulouse (CRCT), Toulouse, France; 6 Université Paul Sabatier, Toulouse III, Toulouse, France; 7 U981, Molecular Predictors and New Targets in Oncology, INSERM, Gustave Roussy, Université Paris-Saclay, 94805 Villejuif, France; 8 Department of Child and Adolescent Oncology, Gustave Roussy, Villejuif, France; 9 Department of Neuropathology, Institute of Pathology, University Hospital Heidelberg, Heidelberg, Germany; 10 Clinical Cooperation Unit Neuropathology, German Consortium for Translational Cancer Research (DKTK), German Cancer Research Center DKFZ, Heidelberg, Germany; 11 Department of Pathology and Neuropathology, GHE, Hospices Civils de Lyon, Lyon, France; 12 Department of Neurosurgery, GHU Paris-Psychiatrie et Neurosciences, Sainte-Anne Hospital, Paris, France; 13 Department of Pathology, Tours Hospital, Tours, France; 14 Department of Pathology, Angers Hospital, Angers, France; 15 Department of Oncology, Pontchaillou Hospital, Rennes, France; 16 Institute of Pediatric Hematology and Oncology (IHOPe), Centre Léon Bérard, Lyon, France; 17 Department of Pediatric Oncology, Institut Curie, Paris, France; 18 Gustave Roussy Cancer Center and Paris-Saclay University, «Genomics and Oncogenesis of Pediatric Brain Tumors» INSERM U981, Villejuif, France; 19 Pediatric Radiology Department, AP-HP, Hôpital Universitaire Necker-Enfants Malades, 75015 Paris, France; 20 Université de Paris, INSERM ERL UA10, INSERM U1163, Institut Imagine, 75015 Paris, France; 21 Department of Biochemistry and Oncogenetics, Paul Brousse Hospital, 94804 Villejuif, France; 22 Department of Pediatric Neurosurgery, Necker Hospital, APHP, Université Paris Descartes, Sorbonne Paris Cité, Paris, France

**Keywords:** Ependymoma, ZFTA, RELA, DNA-methylation, Clusters

## Abstract

The cIMPACT-NOW Update 7 has replaced the WHO nosology of “ependymoma, *RELA* fusion positive” by “Supratentorial-ependymoma, *C11orf95*-fusion positive”. This modification reinforces the idea that supratentorial-ependymomas exhibiting fusion that implicates the *C11orf95* (now called *ZFTA*) gene with or without the *RELA* gene, represent the same histomolecular entity. A hot off the press molecular study has identified distinct clusters of the DNA methylation class of *ZFTA* fusion-positive tumors. Interestingly, clusters 2 and 4 comprised tumors of different morphologies, with various *ZFTA* fusions without involvement of *RELA.* In this paper, we present a detailed series of thirteen cases of non-*RELA ZFTA*-fused supratentorial tumors with extensive clinical, radiological, histopathological, immunohistochemical, genetic and epigenetic (DNA methylation profiling) characterization. Contrary to the age of onset and MRI aspects similar to *RELA* fusion-positive EPN, we noted significant histopathological heterogeneity (pleomorphic xanthoastrocytoma-like, astroblastoma-like, ependymoma-like, and even sarcoma-like patterns) in this cohort. Immunophenotypically, these NFκB immunonegative tumors expressed GFAP variably, but EMA constantly and L1CAM frequently. Different gene partners were fused with *ZFTA*: *NCOA1/2*, *MAML2* and for the first time *MN1*. These tumors had epigenetic homologies within the DNA methylation class of ependymomas-RELA and were classified as satellite clusters 2 and 4. Cluster 2 (n = 9) corresponded to tumors with classic ependymal histological features (n = 4) but also had astroblastic features (n = 5). Various types of *ZFTA* fusions were associated with cluster 2, but as in the original report, *ZFTA:MAML2* fusion was frequent. Cluster 4 was enriched with sarcoma-like tumors. Moreover, we reported a novel anatomy of three *ZFTA:NCOA1/2* fusions with only 1 ZFTA zinc finger domain in the putative fusion protein, whereas all previously reported non-*RELA ZFTA* fusions have 4 ZFTA zinc fingers. All three cases presented a sarcoma-like morphology. This genotype/phenotype association requires further studies for confirmation. Our series is the first to extensively characterize this new subset of supratentorial *ZFTA*-fused ependymomas and highlights the usefulness of *ZFTA* FISH analysis to confirm the existence of a rearrangement without *RELA* abnormality.

**Supplementary Information:**

The online version contains supplementary material available at 10.1186/s40478-021-01238-y.

## Background

Ependymomas (EPN) are glial neoplasms that affect mainly children and young adults. New insights in the genomic and epigenetic landscape of EPN has led to the identification of different groups accordance to their anatomic location (supratentorial, posterior fossa and spinal) [1]. Three subgroups have been identified among supratentorial tumors (ST-EPN): subependymomas; EPN, *YAP1* fusion-positive; and EPN, *RELA* fusion-positive (according to the World Health Organization—WHO—2016 classification) [1–4]. Infrequently (6.5% of cases in one series) [4], *C11orf95* or *RELA* genes have fused with other genes as a result of chromothripsis. The Consortium to Inform Molecular and Practical Approaches to CNS Tumor Taxonomy (c-IMPACT NOW) Update 7 recently proposed the nosology “ST-EPN, *C11orf95-*fusion positive” instead of “ST-EPN, *RELA-*fusion positive” [5]. This modification reinforces the idea that when ST-EPN exhibits fusion that implicates the *C11orf95* gene with or without the *RELA* gene, it represents the same histomolecular entity [4, 6–8]. In recent papers, the methylation classifier based on Forest plot random classification highighted that cases with *C11orf95-*fusion without *RELA* presented epigenetic vicinity with tumors of the EPN-RELA methylation class (MC) and subdivided them into two satellite clusters (2 and 4) by multidimensional reductionality (more specifically t-Distributed Stochastic Neighbor Embedding (t-SNE) analysis) [8, 9]. However, these alternative partners to *RELA* seem to produce original morphological patterns which challenge the histopathological diagnosis. In fact, recent studies have reported a large spectrum of morphologies, including glial, glioneuronal, embryonal and even mesenchymal and epithelial patterns in tumors harboring *C11orf95-*fusions without *RELA* [6, 7, 9]. In this study, we performed a clinico-pathological and molecular analysis (including DNA-methylation profiling and the identification of the new clusters of methylation) of 13 new cases of ST-EPN with *C11orf95* (now called *ZFTA* for Zing Finger Translocation Associated by the new HUGO gene Nomenclature Committee) fusion without the *RELA* gene to more suitably characterize these tumors and compare them with their counterparts which have classical *ZFTA:RELA* fusion.

## Methods

### Study design, patients, data collection

This study included patients diagnosed with ST EPN or glial ST tumors with *ZFTA* rearrangement but no *RELA* rearrangement during ependymal cell differentiation, determined by FISH analyses (techniques previously described [3]).

Epidemiological data (gender and age at diagnosis) and tumor- and treatment-related data (location of tumor and extension, extent of resection, relapses and complementary treatments) were retrospectively analyzed. The extent of the initial resection was assessed by magnetic resonance imaging (MRI) or computed tomography performed after surgery. All the patients’ parents or legal guardians signed informed consent forms before treatment was started. We obtained human subjects approval from our institutional review board.

### Statistical analyses

Unadjusted survival curves for overall survival (OS) and progression-free survival (PFS) were plotted using the Kaplan–Meier method and log-rank tests were used to assess the significance of group comparison. A *p* value of less than 0.05 was considered significant. Statistical analyses were performed using JMP software (version 14.3.0, SAS Institute Inc, Cary, USA). We pooled our data with that of previously reported cases of ST non-*RELA ZFTA*-fused EPN [6–8] and compared them to the data in the literature concerning known EPN with *ZFTA:RELA* fusion, and the other histopathological differential diagnoses such as EPN, *YAP1*-fusion positive, HGNET-*BCOR,* and HGNET-*MN1* [2, 3, 10–22].

### Central radiological review

The central radiological review was performed by two neuroradiologists (NB and VDR). Preoperative MRIs were read and the following features were analyzed: location, tumor size, signal in a T1-weighted sequence and a T2-weighted sequence, susceptibility imaging, the diffusion and apparent diffusion coefficient map (ADC), enhancement, presence of cysts, necrosis, and perfusion parameters.

### Central histopathological review

The central pathology review was performed conjointly by two neuropathologists (ATE and PV).

### Immunohistochemistry

Unstained 3-μm-thick slides of formalin-fixed, paraffin-embedded tissues were obtained and submitted for immunostaining with an automated stainer (Dako Omnis, Glostrup, Denmark). The following primary antibodies were used: CD56 (pre-diluted, clone 123C3, Dako, Glostrup, Denmark), Glial Fibrillary Acidic Protein (GFAP) (1:200, clone 6F2, Dako, Glostrup, Denmark), Olig2 (1:500, clone OLIG2, Sigma-Aldrich, Saint-Louis, USA), vimentin (1:800, clone V9, Dako, Glostrup, Denmark), neurofilament (1:100, clone NF70, Dako, Glostrup, Denmark), NeuN (1:1000, clone A60, Sigma-Aldrich, Saint-Louis, USA), synaptophysin (1:150, clone Synap, Dako, Glostrup, Denmark), EMA (1:200, clone GM008, Dako, Glostrup, Denmark), CK18 (1:200, clone 6F2, Dako, Glostrup, Denmark), smooth muscle actin (1:4000, clone 1A4, Dako, Glostrup, Denmark), NFκB (1:6000, clone D14E12, Cell Signaling Technology, Danvers, USA), L1CAM (1:500, clone UJ127.11, Sigma-Aldrich, Saint-Louis, USA), and Ki-67 (1:200, clone MIB-1, Dako, Glostrup, Denmark). Reticulin staining was performed using the Reticulin silver plating kit according to Gordon & Sweets (Merck Millipore, Guyancourt, France). External positive and negative controls were used for all antibodies and staining.

### FISH analyses

A FISH study was performed on interphase nuclei according to the standard procedures and the manufacturer’s instructions. The *CDKN2A* gene copy number was assessed using the following centromeric and locus specific probes: Vysis CDKN2A/CEP9 FISH Probe Kit (Abbott Molecular, USA).

Deletion was considered if they were detected in more than 30% of nuclei respectively. Results were recorded using a DM600 imaging fluorescence microscope (Leica Biosystems, Richmond, IL) fitted with appropriate filters, a CCD camera, and digital imaging software from Leica (Cytovision, v7.4).

### DNA sequencing

Mutations for the *hTERT* promoter was developed using Massarray iPlex technology and Massarray online design tools (Agena Bioscience) as previously described [23].

### RNA sequencing

RNA was isolated from FFPE (Formalin-fixed paraffin-embedded) tissues with sufficient tumoral density. RNA was extracted using the High Pure FFPET RNA Isolation Kit (catalogue # 06650775001 Roche diagnostics GmbH) according to the manufacturer’s instructions. The RNA concentrations were measured on a Qubit 4 Fluorometer (# Q33238, Thermo Fisher Scientific) with the Invitrogen Qubit RNA BR Kit (# Q10210, Thermo Fisher Scientific). The percentage of RNA fragments > 200 nt (fragment distribution value; DV200) was evaluated by capillary electrophoresis (Agilent 2100 Bioanalyzer). DV200 > 30% was required to process the next steps in the analysis. NGS-based RNA sequencing was performed using the Illumina TruSight RNA Fusion Panel on a Nextseq550 instrument according to the manufacturer’s instructions (Illumina, San Diego, CA, USA). This targeted RNA sequencing panel covers 507 fusion-associated genes, to assess the most recognized cancer-related fusions. The TruSight RNA fusion panel gene list is available at https://www.illumina.com/content/dam/illumina-marketing/documents/products/gene_lists/gene_list_trusight_rna_fusion_panel.xlsx. 7690 exonic regions are targeted with 21,283 probes. Libraries were prepared according to the Illumina instructions for the TruSight RNA fusion Panel kit. STAR_v2.78a and Bowtie software were used to produce aligned readings in relation to the Homo Sapiens Reference Genome (UCSC hg19). Manta v1.4.0, Tophat2 and Arriba v2.1.0 tools were used for fusion calling.

### RT-PCR and Sanger sequencing

RT-PCR: 1 µg of total RNA was retrotranscribed with the primeScript RT Reagent kit (# RR037A, TAKARA). RT-PCR was performed using the Type-it HRM PCR Kit (# 206544, Qiagen GmbH). The primer pairs used for the MN1-C11orf95 fusion confirmation RNASeq results by qPCR were: MN1-F1: 5′-CCTGGGAGAAGGCCAAACC-3′, C11orf95-R1: 5′-CCCCAGGACCCCAAGGCA-3′ (Amplicon size = 85 pb) and the primer pairs used for the MN1-C11orf95 fusion confirmation RNASeq results by Sanger were: MN1-F3: 5′-GCACCATTGACCTGGACTCG-3′, C11orf95-R3: 5′-GGCCTCACAGTGGTCTG-3′ (Amplicon size = 266pb). Amplification conditions were 95 °C—5 min (95 °C—10 s/60 °C—30 s/72 °C—10 s) for 45 cycles. PCRs were performed on a Rotor Gene Q (Qiagen GmbH).

### DNA methylation profiling

Tumor DNA was extracted from freshly frozen tissue samples using the Qiagen DNeasy Blood & Tissue Kit (Cat NO./ID 69504) according to the manufacturer’s instructions. 500 ng of DNA were extracted from each tissue sample. DNA was sent to the Genotyping facility at the German Cancer Research Center (Heidelberg, Germany). All patient samples were analyzed using either Illumina Infinium Methylation EPIC or HumanMethylation450 BeadChip arrays according to the manufacturer’s instructions. Affiliation predictions were obtained from a DNA methylation-based classification web platform for central nervous system tumors (www.molecularneuropathology.org, version 11b4). Next, a t-SNE analysis was performed and compared with the genome-wide DNA methylation profiles from the brain tumor reference cohort [24] as well as with a previous series of *ZFTA:RELA*-fused EPN [3] and with the series of ZFTA-fused ependymomas reported by Zheng et al*.* [9]. Data was generated at the DKFZ Genomics and Proteomics Core Facility (Heidelberg, Germany) as previously described [24].

## Results

### Clinical and radiological characteristics

**Fig. 1:**
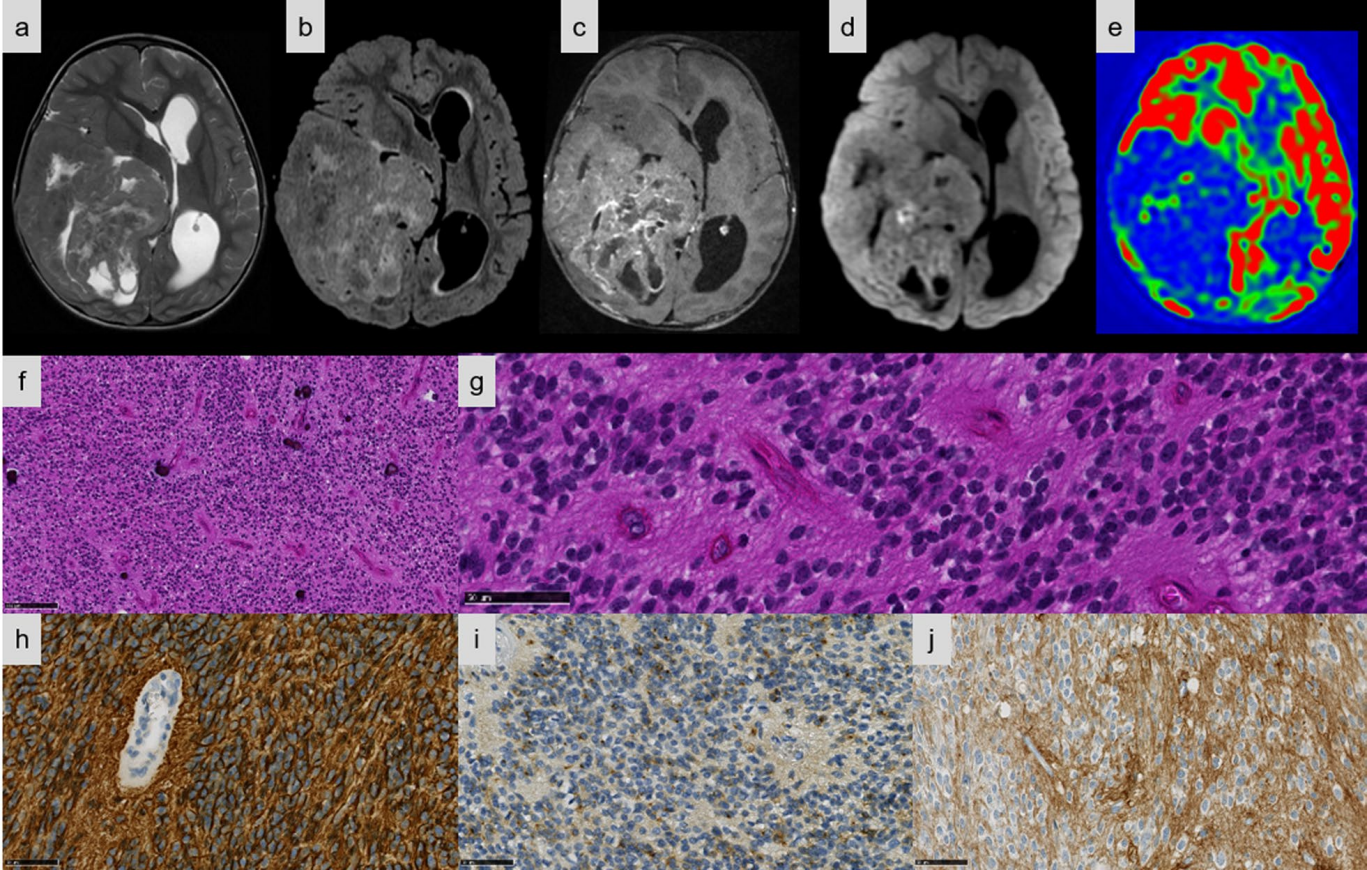
Imaging and histopathological features of Case #8. **a–e** Right temporo-parietal mass, mostly tissular with central necrosis. **a** Peripheral cysts on T2-weighted sequence. **b** Cyst content isointense on FLAIR image. **c** Mild heterogeneous contrast enhancement on T1-weighted sequence after gadolinium injection. **d** Diffusion restriction. **e** Heterogeneous tumoral blood flow with low and intermediate flow areas on Arterial Spin Labelling perfusion imaging. **f** Classical ependymoma-like pattern with calcifications (HPS, magnification × 100). **g** Tumor with perivascular pseudorosettes (HPS, magnification × 400). **h** Tumor with diffuse cytoplasmic immunoexpression of GFAP (magnification × 400). **i** A dot-like pattern of staining for EMA in the tumor (magnification × 400). **j** Diffuse staining for L1CAM (magnification × 400). Black scale bars represent 250 μm (**f**) and 50 µm (**g**–**j**). HPS: Hematoxylin Phloxin Saffron.

**Fig. 2:**
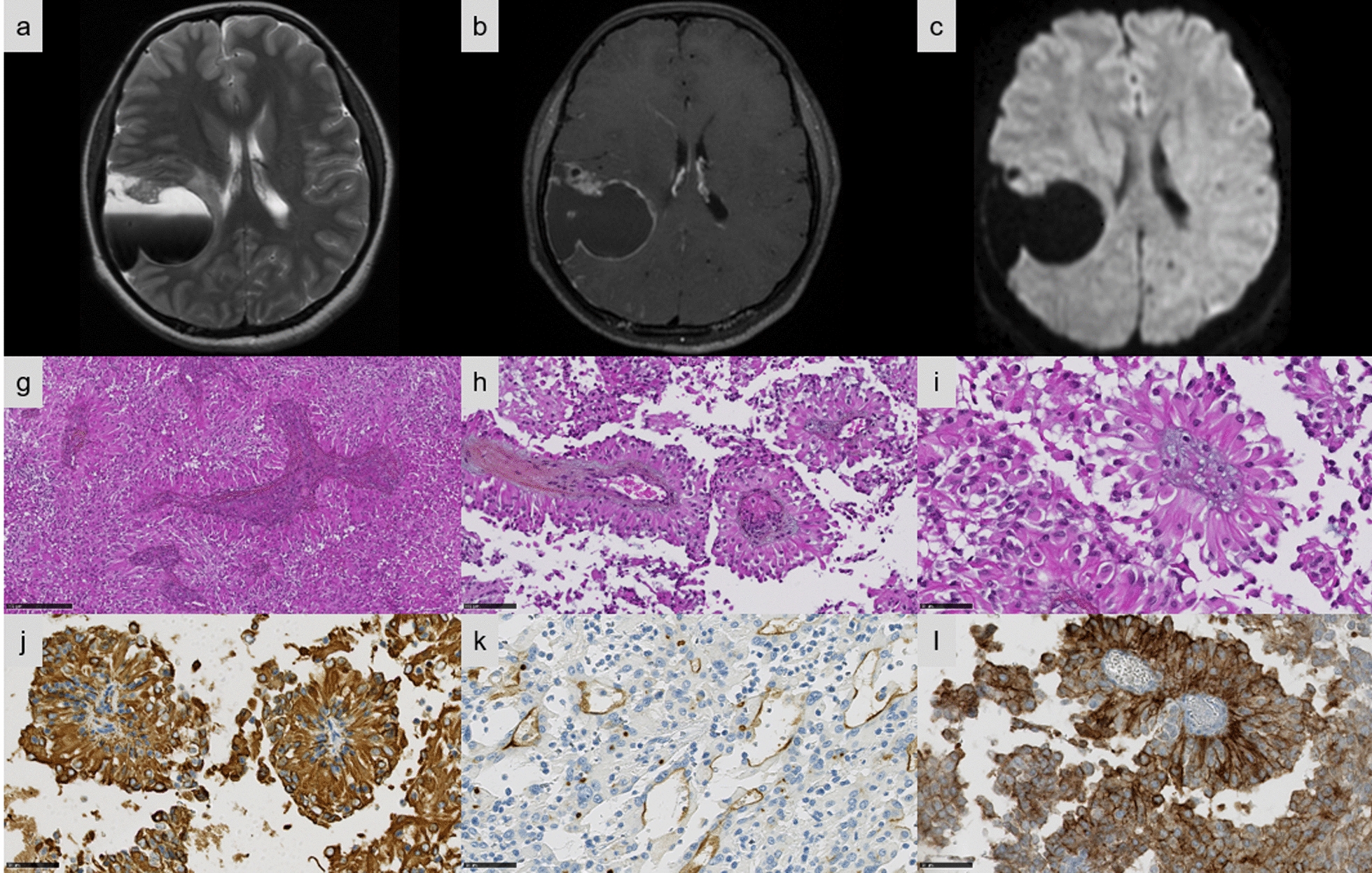
Imaging and histopathological features of Case #3. **a–c** Right frontotemporal cystic mass with thick walls. **a** Cyst fluid–fluid level on T2-weighted sequence. **b** Intense contrast enhancement of the tissular part on T1-weighted sequence after gadolinium injection. **c** Diffusion restriction. **d** Astroblastoma-like pattern composed of multiple pseudorosettes (HPS, magnification × 100). **e** Pseudorosettes composed of a central vessel and variable sclerosis (HPS, magnification × 200). **f** Perivascular pseudorosettes composed of elongated cells containing an abundant eosinophilic cytoplasm (HPS, magnification × 200). **g** The tumor cells strongly expressed GFAP (magnification × 400). **h** A dot-like and apical pattern of staining for EMA in the tumor (magnification × 400). **i** Diffuse staining for L1CAM (magnification × 400). Black scale bars represent 250 μm (**d**), 100 µm (**e**) and 50 µm (**f**–**i**). HPS: Hematoxylin Phloxin Saffron

**Fig. 3:**
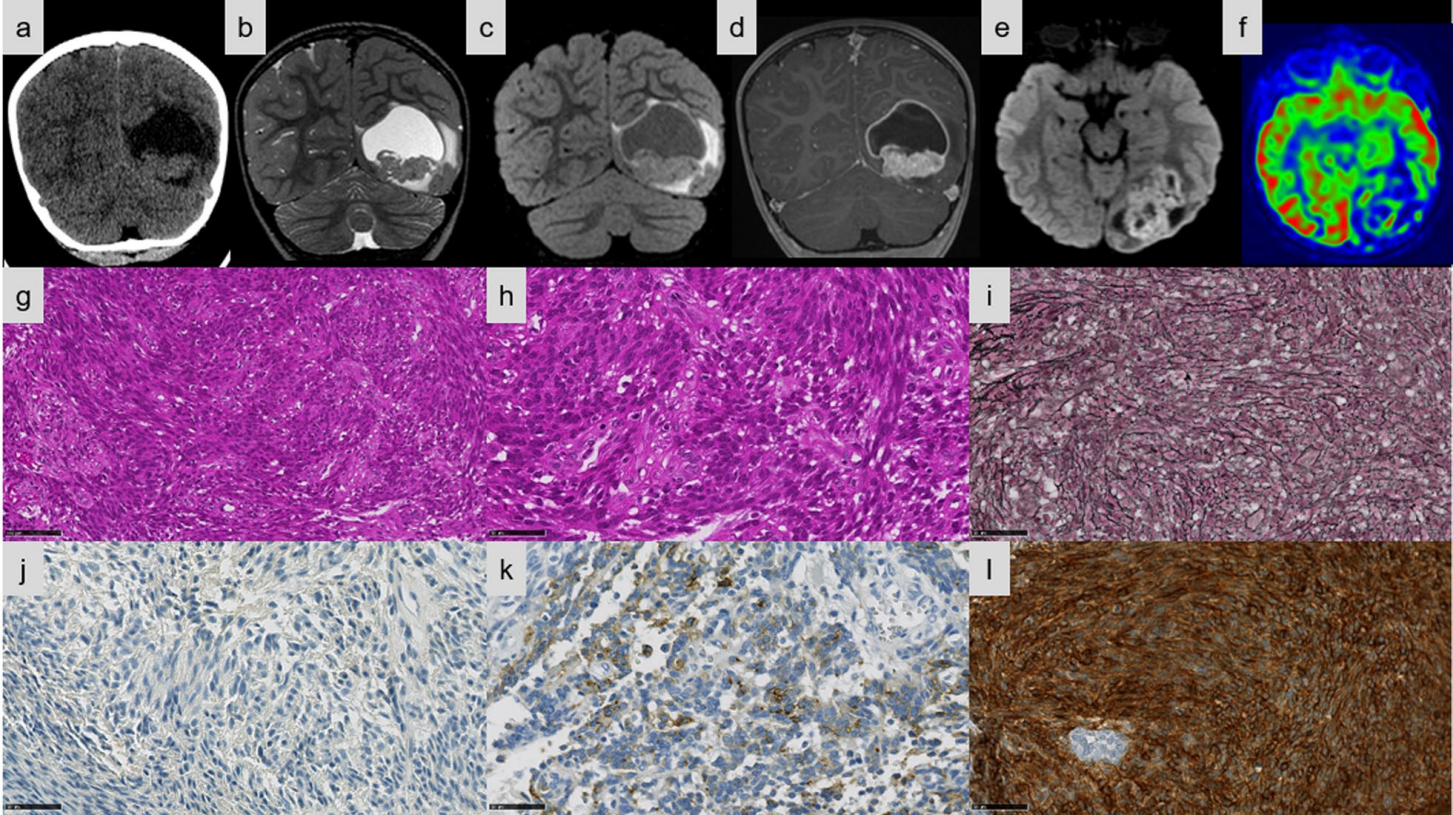
Imaging and histopathological features of Case #11. **a–f** Left occipital cortical cystic mass with tissular mural nodule. **a** Tissular part is slightly hyperdense on CT. **b** peritumoral edema and hyperintense cystic content on T2-weighted sequence. **c** Cyst content isointense on FLAIR image. **d** Intense contrast enhancement on T1-weighted sequence after gadolinium injection. **e** Diffusion restriction. **f** Intermediate tumoral blood flow on Arterial Spin Labelling perfusion imaging. **g** Sarcoma-like pattern composed of fascicles of spindle cells (HPS, magnification × 100). **h** Tumor composed of spindle cells (HPS, magnification × 400). **i** Tumor with a dense reticulin network (magnification × 400). **j** The tumor cells did not express GFAP (magnification × 400). **k** A dot-like and cytoplasmic pattern of staining for EMA in the tumor (magnification × 400). **l** Diffuse staining for L1CAM (magnification × 400). Black scale bars represent 250 μm (**g**) and 50 µm (**h**–**l**). CT: computerized tomodensitometry; HPS: Hematoxylin Phloxin Saffron

**Fig. 4:**
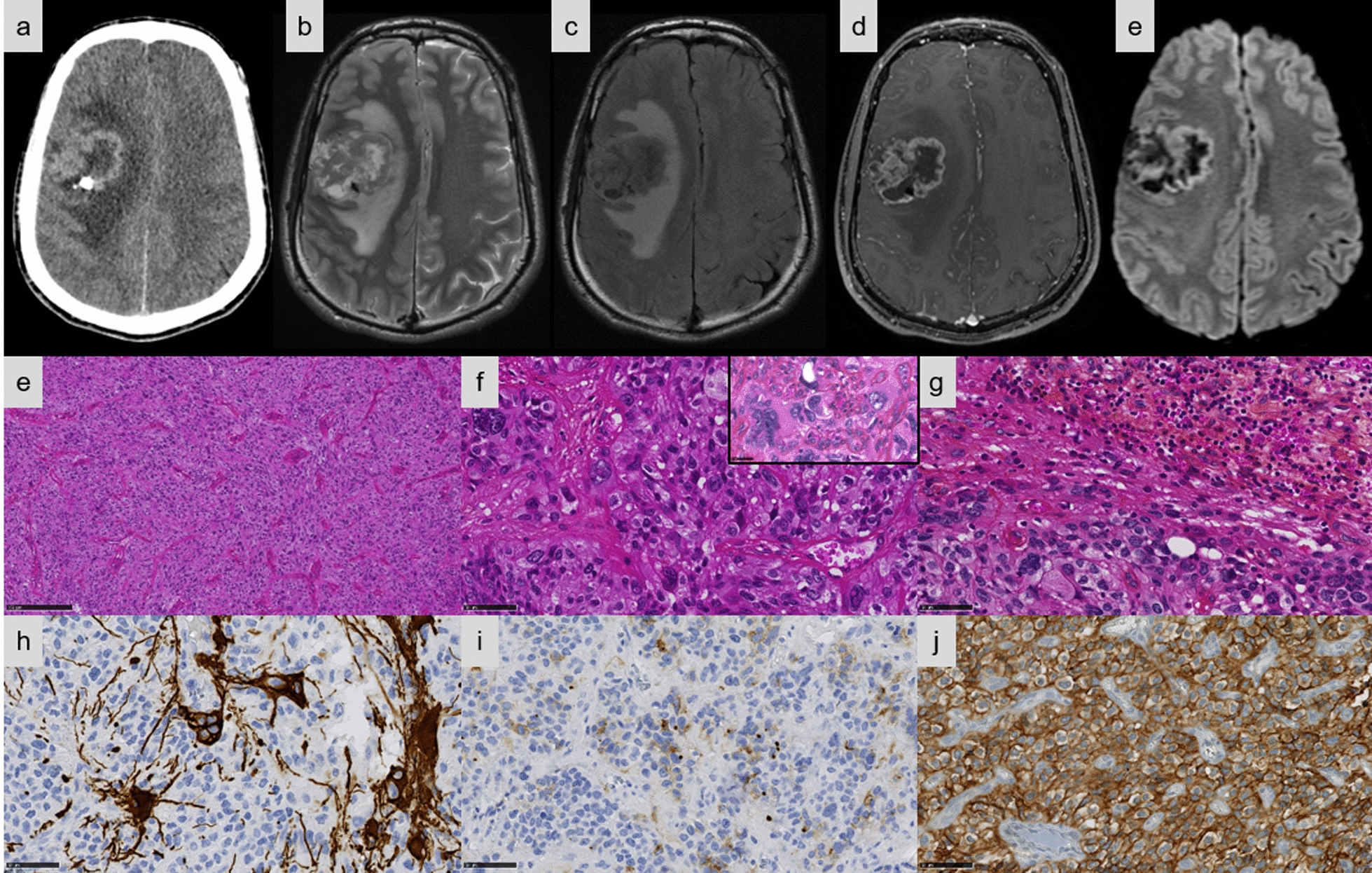
Imaging and histopathological features of Case #10. **a–e** Right frontal cortical tissular mass with necrosis and peripheral cysts, with abundant peritumoral edema. **a** The tissular part is hyperdense on CT with one macrocalcification. **b** Peripheral cysts with abundant peritumoral edema on T2-weighted sequence. **c** Cysts content hypointense on FLAIR image. **d** Intense contrast enhancement on T1-weighted sequence after gadolinium injection. **e** Diffusion restriction. **f** A solid tumor with a rich vascular network (HPS, magnification × 100). **g** Tumor cells with nuclear and cytoplasmic pleomorphism with intranuclear inclusions (HPS, magnification × 400). **h** Some focal collections of lymphocytes (magnification × 400). **i** The proliferation presented a patchy expression for GFAP (magnification × 400). **j** A dot-like and cytoplasmic pattern of staining for EMA in the tumor (magnification × 400). **k** Diffuse staining for L1CAM (magnification × 400). Black scale bars represent 250 μm (**f**) and 50 µm (**g**–**k**). CT: computerized tomodensitometry; HPS: Hematoxylin Phloxin Saffron.

**Fig. 5:**
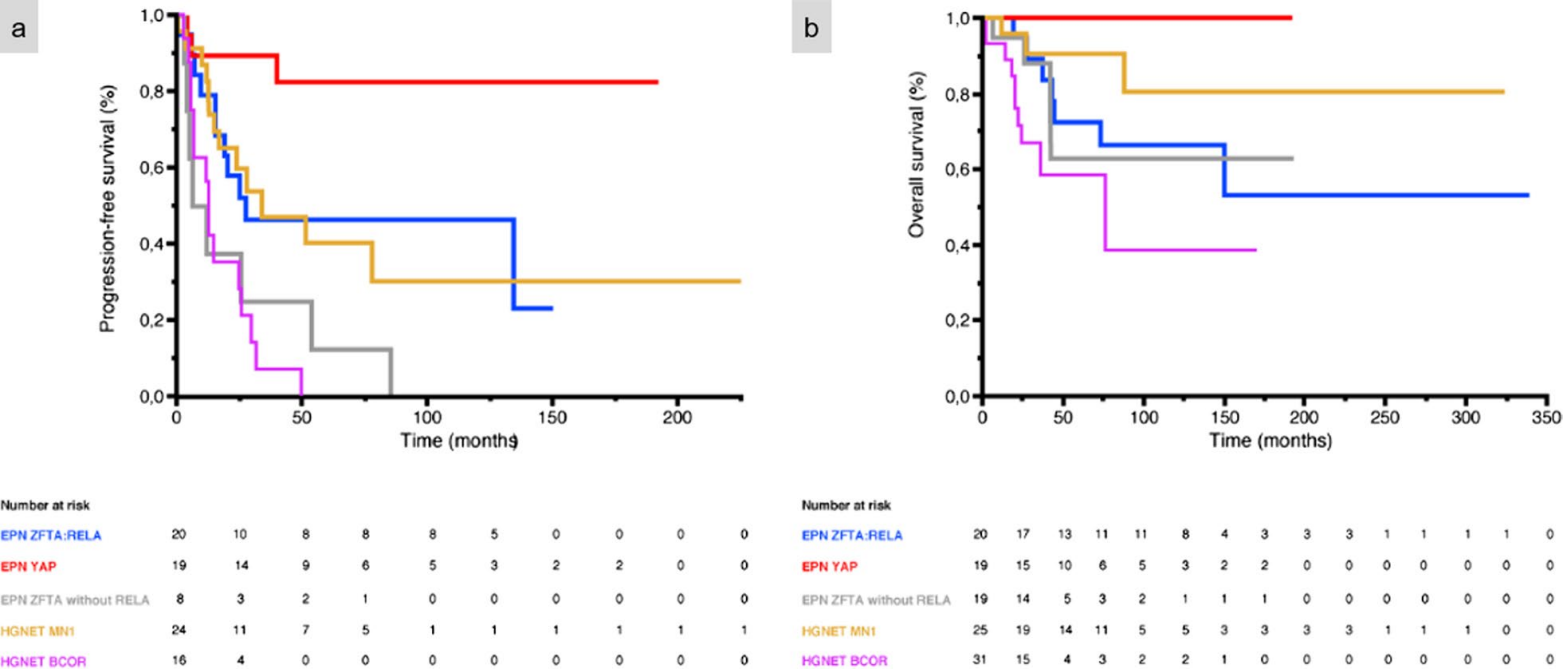
Prognosis for our cases. **a** The mean/median PFS were 70.4/27.6 months for EPN-*RELA*, 36.3/ months not reached for EPN-*YAP*, 24.4/9.2 months for non-*RELA ZFTA*-fused EPN and 43.9/34.0 months for HGNET-*MN1* and 16.2/12.0 for HGNET-*BCOR* with a significant difference in univariate analysis (*p* < 0.001). **b** The median OS was not reached for any of the subgroups except HGNET-*BCOR* (76.0 months) and the mean OS was not reached for the EPN-*YAP* subgroup. The mean OS were 113.5 months for EPN-*RELA*, 39.3 months for non-*RELA ZFTA*-fused EPN, 81.6 months for HGNET-*MN1* and 53.2 months for HGNET-*BCOR* with a significant difference in univariate analysis (*p* = 0.003)

Relevant clinical data are summarized in Table 1. The median age at diagnosis was 6.7 years (patients’ ages ranged from 9 months to 41 years). The male/female sex ratio was 1.6 (8 males and 5 females). Tumor locations varied; the frontal lobe being the most common location (6/13 cases, 46%). Detailed MRIs were available for 12/13 cases (Figs. 1, 2, 3, 4). The size of the tumor ranged from 4 to 12 cm. Nine tumors (Cases #1, 3, 4, 5, 8, 10, 11, 12, and 13) showed a similar imaging pattern: well-demarcated masses, located in the hemispheres with a large cystic portion, and a thick heterogeneous solid component intensely enhanced after gadolinium injection. Peritumoral edema was always present and frequently abundant. Of the six cases with a FLAIR sequence available, two had hyperintense intracystic content (Figs. 1, 4). Conversely, the three remaining cases presented a prominent solid component without cystic content (Cases #6, 7, and 9), mild or no peritumoral edema, and variable contrast enhancement (mild in Cases #6 and 7, intense in Case #9). Diffusion was restricted in 6/8 patients with available sequences (Figs. 1c, 3c), and intermediate in the two remaining cases. Cerebral Blood Flow using Arterial Spin Labelling (ASL) was intermediate (maximal value in the tumor: 50 to 56 mL/min/100 g) in 3/3 cases with available sequences (Figs. 1d, 3d). All patients, except two (Cases #1 and 9) underwent total resection. All patients, except two (Cases #2 and 13), received adjuvant treatment (mainly conventional focal radiation therapy). Outcome data was available for all patients included in the cohort. Six (46%) patients had tumor recurrence, with a mean PFS of 30.1 months (median 16.1 months; CI 95%: 4–85). Two patients (Cases #1 and 9) died of their disease, with a mean OS of 24 months. The two patients who died were those who had not undergone total resection. When we pooled our data with data from the literature*,* the mean/median PFS were 70.4/27.6 months for EPN, *ZFTA:RELA*-fused, 36.3 months/ not reached for EPN, *YAP1*-fusion positive, 24.4/9.2 months for ST non-*RELA ZFTA*-fused EPN*,* 43.9/34.0 months for HGNET-*MN1* and 16.2/12.0 months for HGNET-*BCOR* with a significant difference in all groups on univariate analysis (*p* < 0.001). The median OS was not reached for all subgroups except for HGNET-*BCOR* (76.0 months) and the mean OS was not reached for the EPN, *YAP1-*fusion positive subgroup. The mean OS were 113.5 months for *ZFTA:RELA*-fused EPN, 39.3 months for ST non-*RELA ZFTA*-fused EPN, 81.6 months for HGNET-*MN1* and 53.2 months for HGNET-*BCOR* with a significant difference in all groups on univariate analysis (*p* = 0.003) (Fig. 5). Unlike OS which did not show significant differences, the PFS was significantly different between ST non-*RELA ZFTA*-fused EPN and EPN, *ZFTA:RELA*-fused (*p* = 0.023), EPN, *YAP1*-fusion positive (*p* < 0.001) and HGNET-*MN1* (*p* = 0.036). We found no significant difference between ST non-*RELA ZFTA*-fused EPN and HGNET-*BCOR* (*p* = 0.700).

**Table 1:** Case list of our series of non-*RELA ZFTA* fused-EPN with clinical features

Case	Sex, age	Location	Surgery	Adjuvant treatment	Local recurrence, PFS (mo)	Clinical outcome, OS (mo)
1	F, 5 yo	Right frontal and temporal lobes	PR	CT	Yes, 5	Dead, 42
2	M, 41 yo	Left carrefour	TR	No	Yes, 85	Alive, 193
3	F, 26 yo	Right frontal and temporal lobes	TR	RT	No	Alive, 9
4	M, 11 yo	Left frontal and parietal lobes	TR	RT	No	Alive, 10
5	M, 8 yo	Left frontal lobe	TR	CT + RT	No	Alive, 34
6	M, 9 yo	Right frontal lobe	TR	RT	Yes, 4	Alive, 90
7	M, 1 yo	Left parietal and occipital lobes	TR	PT	No	Alive, 37
8	M, 3 yo	Right temporal and parietal lobes	TR	RT	No	Alive, 28
9	F, 9 mo	Right parietal lobe	PR	CT	Yes, 6	Dead, 6
10	M, 26 yo	Right frontal lobe	TR	CT + RT	No	Alive, 70
11	F, 4 yo	Left occipital lobe	TR	CT + PT	Yes, 25	Alive, 58
12	M, 7 yo	Right temporal, parietal and occipital lobes	TR	RT	No	Alive, 33
13	F, 2 yo	Left parietal and occipital lobes	TR	No	Yes, 53	Alive, 115

CT: chemotherapy; F: female; M: male; mo: months; OS: overall survival; PFS: progression free survival; PR: partial resection; PT: proton therapy; RT: radiation therapy; TR: total resection; yo: years old

### Histopathological and immunohistochemical characterization

Detailed histopathologic and immunohistochemical data are presented respectively in Additional files 1 and 2. The most predominant pattern (5/13 cases) was EPN-like, consisting of well-circumscribed tumors composed of mainly clear cells; with perivascular pseudorosettes, rosettes and delicate branching vessels demonstrating a chicken-wire appearance (Cases #5, 6, 7, 8, and 9) (Fig. 1e–f). Another frequently observed morphological pattern was astroblastoma-like (4/13 cases), consisting of well-demarcated tumors composed of astroblastic pseudorosettes (Cases #1, 2, 3, and 4) (Fig. 2d–f). The third histopathological pattern observed (Cases #11, 12, and 13) was composed of spindle-shaped cells arranged in bundles with a reticulin network in two cases (Cases #11 and 13) (Fig. 3e–f). The last tumor (Case #10) presented a pleomorphic xanthoastrocytoma (PXA)-like morphology, composed of large pleomorphic and multinucleated cells with nuclear inclusions, associated with perivascular lymphocytic infiltrates (Fig. 4e–g). However, we observed no eosinophilic granular bodies or dense reticulin network. Calcifications were a common finding (8/13 cases, regardless of the histopathological pattern). A fibrous collagenous stroma was observed in six cases (Cases #2, 3, 8, 10, 11, and 13). Mitotic counts ranged from 2 to 102 per 10 high-power fields. In all cases except two (Cases #2 and 13), necrosis was observed including only one case of palisading necrosis (Case #8). Microvascular proliferation was present in all cases except one (Case #2). All cases except one (Case #1) exhibited CD56 staining, whereas vimentin was consistently expressed. GFAP immunoreactivity was identified in the cytoplasm and fibrillary processes of tumors with EPN-like, astroblastoma-like features (Figs. 1g, 2g), whereas no immunopositivity or only a focal expression was detected in tumors with sarcoma-like and PXA-like features (Figs. 3h, 4h). Olig2 was focally expressed in most tumors and absent in five tumors (Cases #4, 7, 11, 12, and 13). Neurofilament staining confirmed the solid growth pattern of all cases (except Cases #3 and 10, which were partially infiltrative). All cases were EMA immunopositive with varying patterns (cytoplasmic, membranous, apical, dot-like and with micro-lumens) (Figs. 1h, 2h, 3i, 4i). CK18 immunopositivity was present in 6/13 cases. Neuronal markers were positive in 11/13 cases, often only focally, without ganglion cell differentiation. Nuclear NFκB expression was only observed in a few nuclei in two tumors (Cases #3 and 9), whereas 12/13 cases presented L1CAM immunoexpression with a mixture of varying degrees of distribution and intensity (Figs. 1i, 2i, 3j, 4j). No alpha-smooth muscle actin reactivity was identified in any case. The MIB-1 labelling index ranged from 4 to 70%.

### Molecular results

FISH analyses for *CDKN2A* failed to reveal any deletion in any of the cases tested (n = 13). No mutation of *hTERT* was evidenced in any of the cases tested (n = 13).

**Fig. 6:**
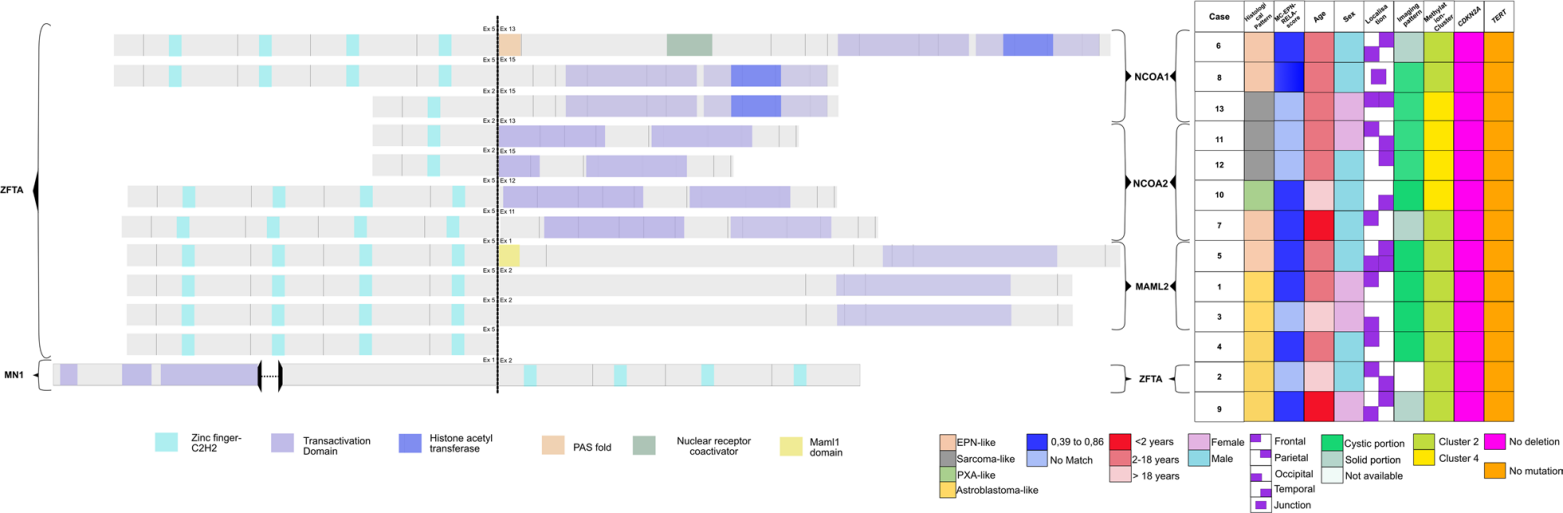
Anatomy of non-*RELA ZFTA*-fusions. The main clinical and histopathological features are indicated for each fusion as well as the corresponding score for the RELA-fusion ependymoma using the DNA methylation-based classification (Heidelberg Brain Tumor Classifier version 11b4)

We found a new *MN1:ZFTA* fusion which was verified by RT-PCR and Sanger sequencing for case #2 (Additional file 3). Other *ZFTA* partners have been previously described [4, 6–9]. The anatomy of the 11 in frame fusions retrieved is illustrated in Fig. 6, including 3 *ZFTA:MAML2* fusions, 3 *ZFTA:NCOA1* fusions and 4 *ZFTA:NCOA2* fusions. In case #4, we found five spanning reads with the ZFTA mid-exon 5 joined to a non-coding intergenic region on chromosome 11 (PGR-AS1(100810), TRPC6(141163)). The putative chimeric ZFTA protein essentially corresponds to a ZFTA protein truncated at its very C-terminal end, because a STOP codon is reached after only a few codons in the 3’partner sequence. Breakpoints are provided in Additional file 4. Detailed chromosomal coordinates are given using hg19. With this Illumina TruSight RNA Fusion Panel, we had only one technical failure out of 13 cases (FFPE block over 8 years old).

**Fig. 7:**
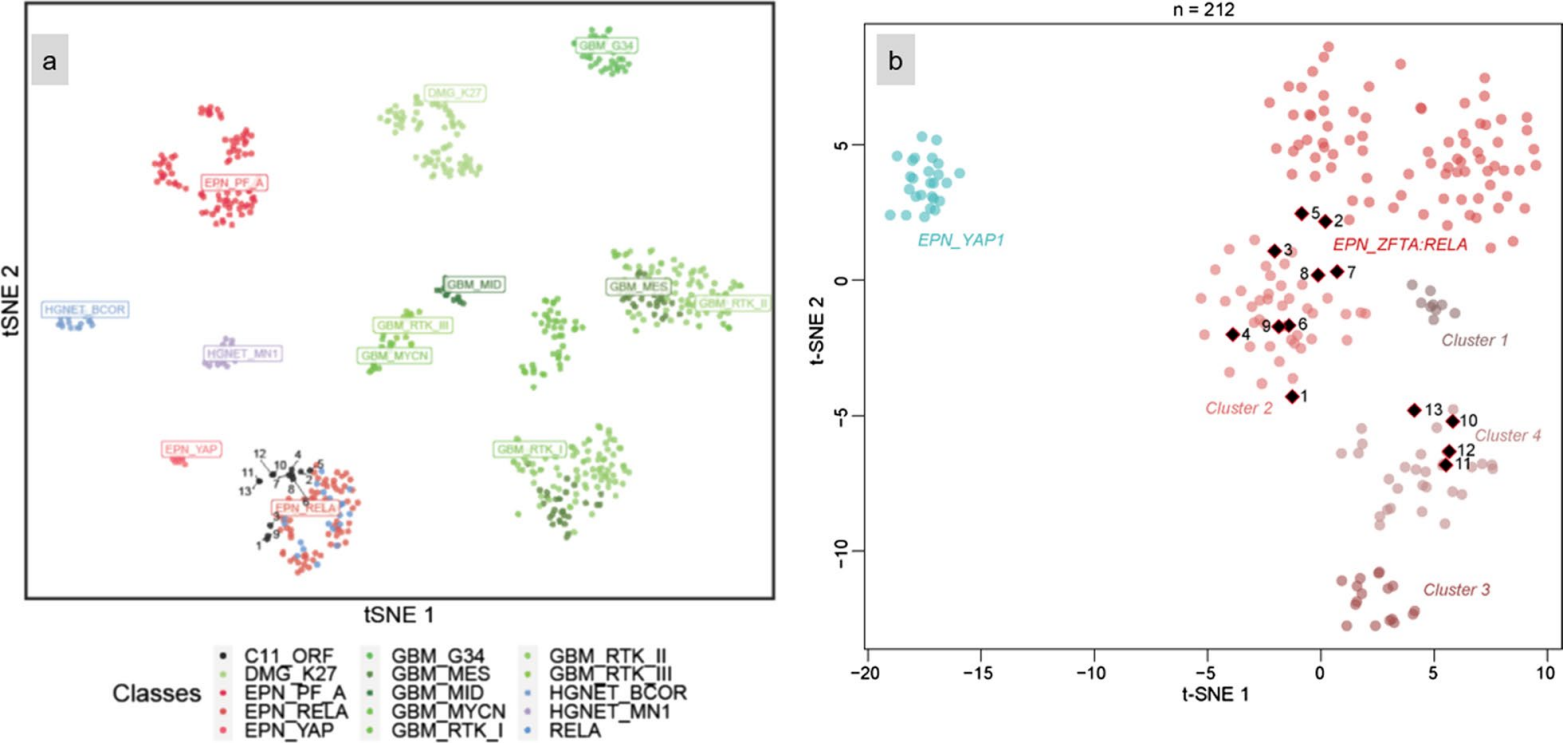
DNA methylation-based t-distributed stochastic neighbor embedding distribution. Our 13 tumors were compared to 600 reference samples from the Heidelberg cohort belonging to the HGNET_*BCOR* (n = 23)*,* HGNET_*MN1* (n = 21), EPN_*RELA* (n = 70), EPN_*YAP* (n = 11) methylation classes which constitute histopathological differential diagnoses, and EPN-PF_A (n = 91), DMG_K27 (n = 78), GBM_MID (n = 14), GBM_RTK_III (n = 13), GBM_MYCN (n = 16), GBM_MES (n = 56), GBM_RTK_I (n = 64), and GBM_RTK_II (n = 143) and our cases previously reported in Pages et al*.* of EPN_RELA with proven *ZFTA:RELA-*fusion (n = 22). The cases in this study, indicated as black dots, were in close proximity to the EPN_RELA subgroup. In a more focused t-SNE analysis of the samples alongside the recently described satellite clusters of *ZFTA*-fusion positive ependymoma and *YAP1*-altered ependymoma, four of the cases grouped with cluster 4 and nine with cluster 2

According to the DNA methylation-based classification and the DKFZ Classifier (version 11b4), none of the tumors were classifiable (calibrated scores for DNA methylation class < 0.9). Although none of the cases received a calibrated score ≥ 0.9 in the current version (11b4) of the CNS tumor classifier, most of the tumors obtained the highest score for ependymal subclasses (EPN-RELA) with valid quality controls for all samples. A t-SNE analysis was performed to compare the genome-wide DNA methylation profiles of our previous EPN-RELA cohort with proven *RELA:ZFTA* fusion (n = 80) [3], EPN-YAP (n = 26), HGNET-*BCOR* (n = 23) and HGNET-*MN1* (n = 21) in the CNS reference cohort [19]. All cases clustered in close proximity to EPN-RELA (Fig. 7). Copy number profiles are detailed in Additional files 5–17. In a more focused t-SNE analysis of DNA methylation data of these samples alongside the recently described satellite clusters of *ZFTA*-fusion positive EPN (cluster 1, n = 9; cluster 2, n = 40; cluster 3, n = 17, and cluster 4, n = 27) [9], four of the cases grouped with cluster 4 and nine with cluster 2 (Fig. 7).

## Discussion

Like ST *ZFTA:RELA*-fused EPN, ST non-*RELA ZFTA*-fused EPN affected mainly children [4, 6–8]. The sex ratio was 1.3 (13 males and 10 females) [4, 6–8]. Radiologically, non-*RELA ZFTA*-fused EPN presented some similarities with their classical counterparts with *ZFTA:RELA* fusion [3]. In fact, they were mainly characterized by well-demarcated solid and cystic lesions with peripheral enhancement of the cystic content [3]. However, contrary to *ZFTA:RELA*-fused EPN, peripheral edema was significant in our cases and for the most part the cystic component was not hyperintense on the FLAIR sequence [3]. ST non-*RELA ZFTA*-fused EPN presented high morphological heterogeneity with only rare cases having histopathological and immunohistochemical features of *ZFTA:RELA-*fused EPN [8, 9]. In the literature, their histological appearance was sarcoma-like, PXA-like, high-grade glioma-like, malignant teratoma-like, embryonal tumor-like, or had neuronal differentiation and a granular cell component [6, 7, 9]. We also identified four cases with astroblastoma-like features. Despite this phenotypical heterogeneity, all tumors were in close epigenetic proximity to the MC EPN-RELA. As expected, our cases with *ZFTA* fusion without *RELA* were subclassified in clusters 2 and 4 [9]. In the original report, the cluster 2 corresponded almost exclusively to tumors with ependymal morphology [9]. In our series, 4/9 tumors in cluster 2 showed ependymal features and 5 presented astroblastoma-like features, noted for the first time. In the original report [9], the tumors in cluster 4 corresponded to highly malignant poorly differentiated tumors including one malignant small-cell sarcomatoid carcinoma and one undifferentiated sarcoma [9]. In our series, all three tumors presenting with sarcoma histology were classified in cluster 4. None of the cases in our series or from those in the literature exhibited significant nuclear expression of NFκB [7, 8], which supports previous studies showing that p65 immunoexpression is highly correlated to the presence of *RELA* fusion [3, 25, 26]. However, all except two cases [6, 7, 9] showed L1CAM immunoexpression to varying degrees and intensities, confirmed by the RNA expression data [4]. Consequently, L1CAM may represent a diagnostic tool for non-*RELA, ZFTA*-fused EPN. Further immunohistochemical series including different molecularly defined CNS entities are needed to draw a conclusion on the sensitivity/specificity of this biomarker. The landscape of gene partners of *ZFTA*-fused EPN (without *RELA*) is wide, the main being *MAML2* (21/51 cases), *NCOA2* (14/51 cases), and *NCOA1* (9/51 cases) genes [6–9]. These fusions alone are sufficient to drive tumorigenesis in vivo [6, 7, 9]. In the original report, the main cases of *ZFTA:MAML2* fusion were in cluster 2 and showed ependymal features [9]. Our data are in line with this report as our three cases with *ZFTA:MAML2* were classified as cluster 2 and showed a histological phenotype of EPN but also of astroblastoma. A *MN1:ZFTA* fusion with *ZFTA* as a 3’ partner was noted for the first time in another of our cases, as was previously reported in one case with *LTBP3:ZFTA* fusion [4]. Interestingly, this case of *MN1:ZFTA* fusion presented astroblastoma-like features, but was in close vicinity of the MC EPN-RELA (cluster 2) and not HGNET-*MN1*. The *MN*1 breakpoint is similar to that of the *MN1:BEND2* fusion, which could constitute a diagnostic pitfall if only the MN1 breakapart FISH is used. We found a *ZFTA* fusion with a non-coding region which probably leads to a truncated ZFTA protein at the C terminal end. At the mRNA level, the truncation of 3’UTR of ZFTA eliminates three miRNA binding sites (hsa-miR-424-3p URS00002BCF86_9606) involved in regulating ZFTA expression. This loss of regulation could lead to a nuclear accumulation of ZFTA sustaining oncogenicity with a cis-acting mechanism instead of the trans-activating mechanism that could not take place in the absence of a coactivating partner. This cis-acting mechanism has been suggested by Zhu et al*.* because only half of the top-scoring ChiP-seq peaks of ZFTA-RELA chimeric proteins containing one or more ZFTA DNA binding motifs [27]. They hypothesized that the no-motif peaks might be bound by a ZFTA-RELA-containing protein complex that uses another pioneer subunit to initiate chromatin binding, necessitating a cis-acting mechanism. In our case, this ZFTA truncation led to an astroblastic phenotype that could correspond to the purely oncogenic cis-effect of ZFTA. This cis-acting hypothesis should be tested in mechanistic studies that are beyond the scope of our descriptive study. It is interesting to note that in our series with detailed histological typing, none of the *ZFTA:NCOA1/2* fusions showed any astroblastoma phenotype, which highlights the potential role of the ZFTA partner in the histological phenotype. However, the number of ZFTA zinc fingers is also important. Previous studies have shown that the number of ZFTA zinc fingers in *ZFTA:RELA* fusions impact oncogenicity as well as the chromatin binding sites [28]. In *ZFTA:RELA* fusions, the 5’ZFTA part of the chimeric protein has one (RELAfus1) or two (RELAfus2) zinc finger domains while RELA is consistently almost full length. Parker et al*.* showed that neural stem cells (NSCs) transduced with RELAfus 1 generated tumors after intra-cerebral implantation in nude mice [4]. RELAfus2 NSCs also generated tumors albeit with clearly lower lethality. Using ChiP sequencing, Zhu et al*.* showed that chimeric protein RELAfus1 binds to 32,135 binding sites, RELAfus2 to 13,954 with only 5338 common sites with fus1 [27]. Therefore, the number of zinc finger domains in the ZFTA fusion necessarily impact the tumoral biology together with the fusion partner. Four ZFTA zinc finger domains is the rule in cases of non-*RELA ZFTA*-fused EPN published so far [6–9] and this was present in ten of our cases. However, whereas four *ZFTA:NCOA1/2* with four zinc fingers corresponded to three classical ependymal histomorphologies and one PXA-like morphology, all three of our *ZFTA:NCOA1/2* fusions with only one zinc finger corresponded to a sarcoma-like phenotype. We are the first to report such cases and it remains to be confirmed whether a sarcoma phenotype together with a cluster 4 of ST EPN is consistently related to this type of fusion. Retrospective molecular studies of tumors initially diagnosed as primitive CNS sarcomas would be useful to answer this question. Our study and the data in the literature indicate that the outcome is the worst for patients with ST non-*RELA ZFTA*-fused EPN. When the PFS and OS are compared with their *ZFTA:RELA-*fused counterparts, we noticed a significant difference between the two ST EPN subgroups for PFS but not for OS. However, these results are limited by the low number of reported cases and further studies concerning the prognosis and histopathologic phenotype of the ST *ZFTA-*fused EPN subgroups are required.

In conclusion, our series characterizes a cohort of ST non-*RELA ZFTA*-fused EPN which present a large spectrum of histopathological features, including some overlapping with, *ZFTA:RELA*-fused EPN and poorly differentiated tumors with sarcoma-like features. Despite this heterogeneity, DNA methylation profiling confirmed their epigenetic proximity to the MC EPN-RELA and were subclassified in clusters 2 and 4. Regardless of the morphology, EMA and L1CAM immunopositivity (without NFκB expression) in ST tumors may incite neuropathologists to suggest this diagnosis. Our work highlighted the usefulness of *ZFTA* FISH analysis to confirm the existence of a rearrangement without *RELA* abnormality.

## Supplementary Information

**Additional file 1**.

**Additional file 2**.

**Additional file 3**. Sanger sequencing of RT-PCR products for MN1:ZFTA fusion.

**Additional file 4**.

**Additional file 5**. Copy number variation profile of case #1.

**Additional file 6**. Copy number variation profile of case #2.

**Additional file 7**. Copy number variation profile of case #3.

**Additional file 8**. Copy number variation profile of case #4.

**Additional file 9**. Copy number variation profile of case #5.

**Additional file 10**. Copy number variation profile of case #6.

**Additional file 11**. Copy number variation profile of case #7.

**Additional file 12**. Copy number variation profile of case #8.

**Additional file 13**. Copy number variation profile of case #9.

**Additional file 14**. Copy number variation profile of case #10.

**Additional file 15**. Copy number variation profile of case #11.

**Additional file 16**. Copy number variation profile of case #12.

**Additional file 17**. Copy number variation profile of case #13.

## Abbreviations

DMG: Diffuse midline glioma
EPN: Ependymoma
GBM: Glioblastoma
HGNET: High-grade neuroepithelial tumor
MES: Mesenchymal
MID: Midline
PF_A: Posterior fossa subgroup A
