# Supplementary figures and images for: Supratentorial non-RELA, ZFTA-fused ependymomas: a comprehensive phenotype genotype correlation highlighting the number of zinc fingers in ZFTA-NCOA1/2 fusions
Source: Acta Neuropathol Commun. 2021 Aug 13;9:135. doi: 10.1186/s40478-021-01238-y (PMC8362233; doi:10.1186/s40478-021-01238-y)

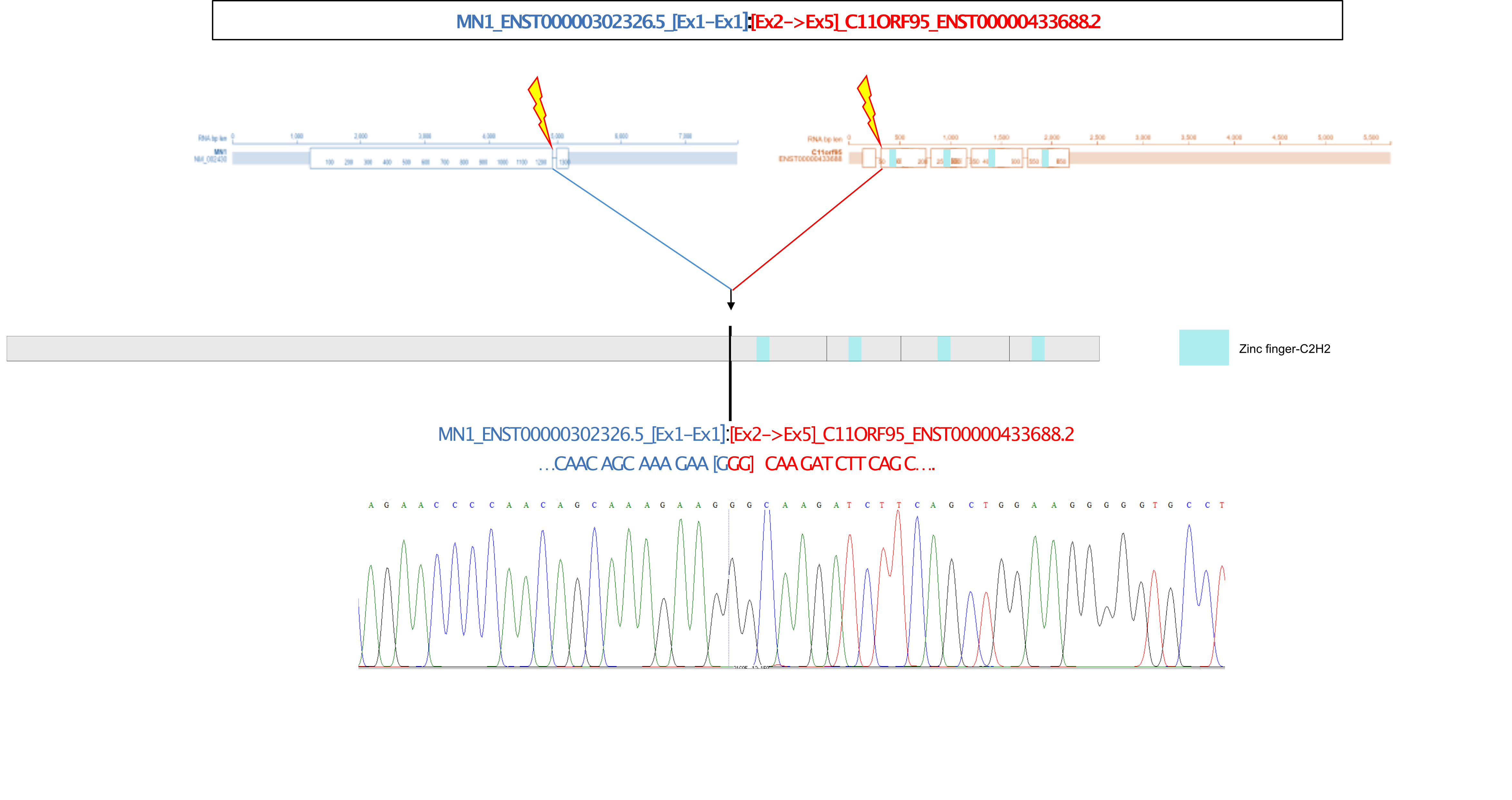

Supplement: Supplementary file 3 — Additional file 3. Sanger sequencing of RT-PCR products for MN1:ZFTA fusion. [file 40478_2021_1238_MOESM3_ESM.tiff]

Case\_1

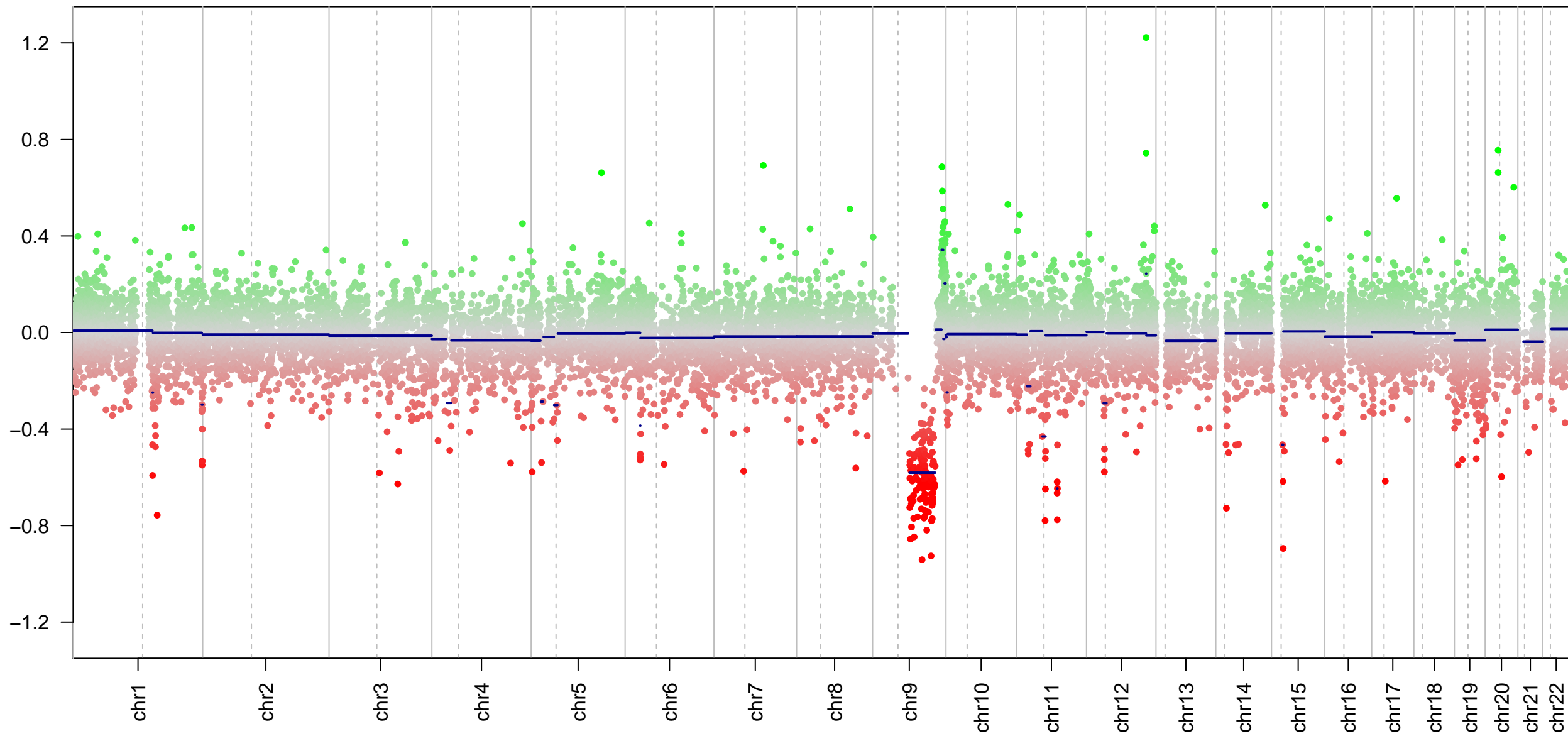

Supplement: Supplementary file 5 — Additional file 5. Copy number variation profile of case #1. [file 40478_2021_1238_MOESM5_ESM.pdf]

Case\_2

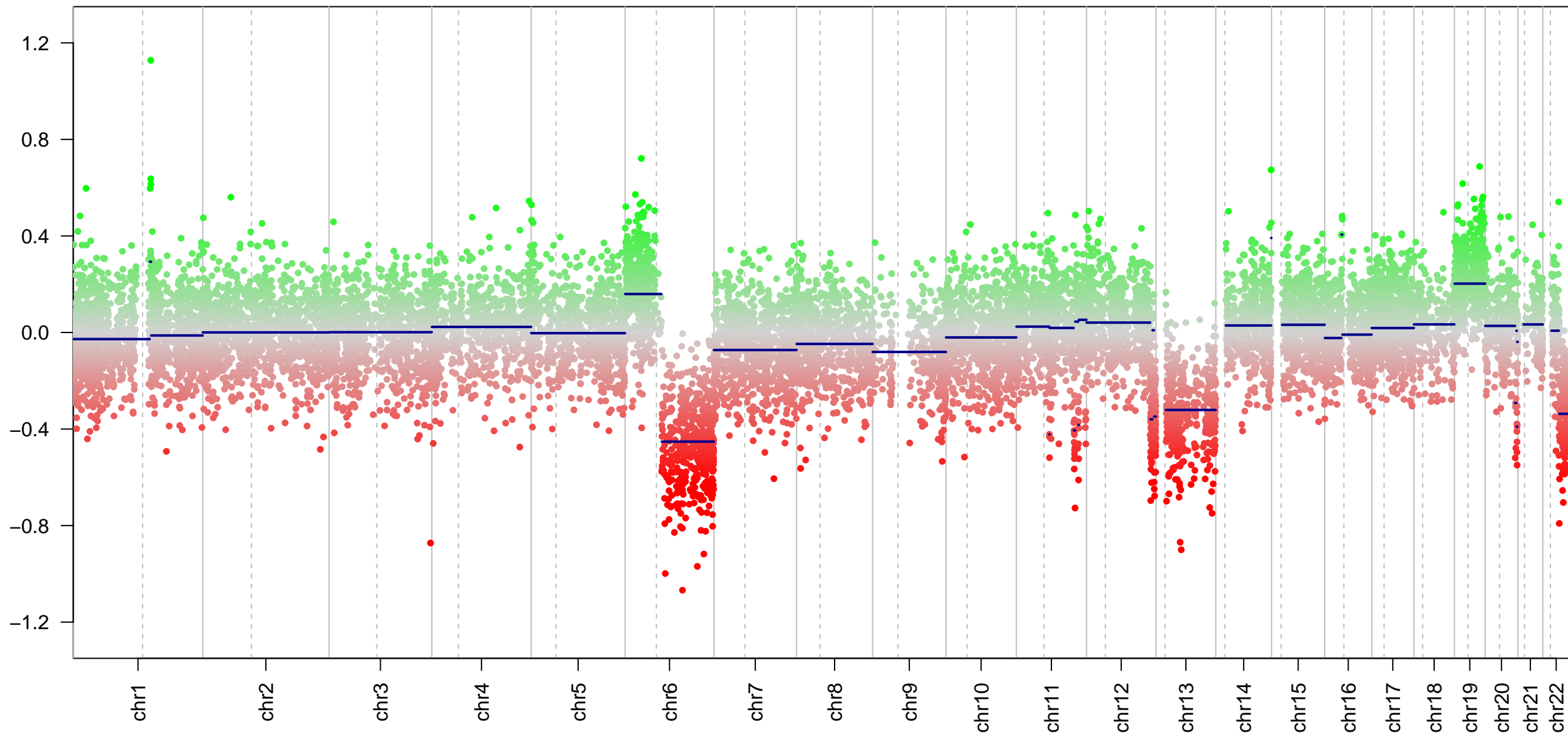

Supplement: Supplementary file 6 — Additional file 6. Copy number variation profile of case #2. [file 40478_2021_1238_MOESM6_ESM.pdf]

Case\_3

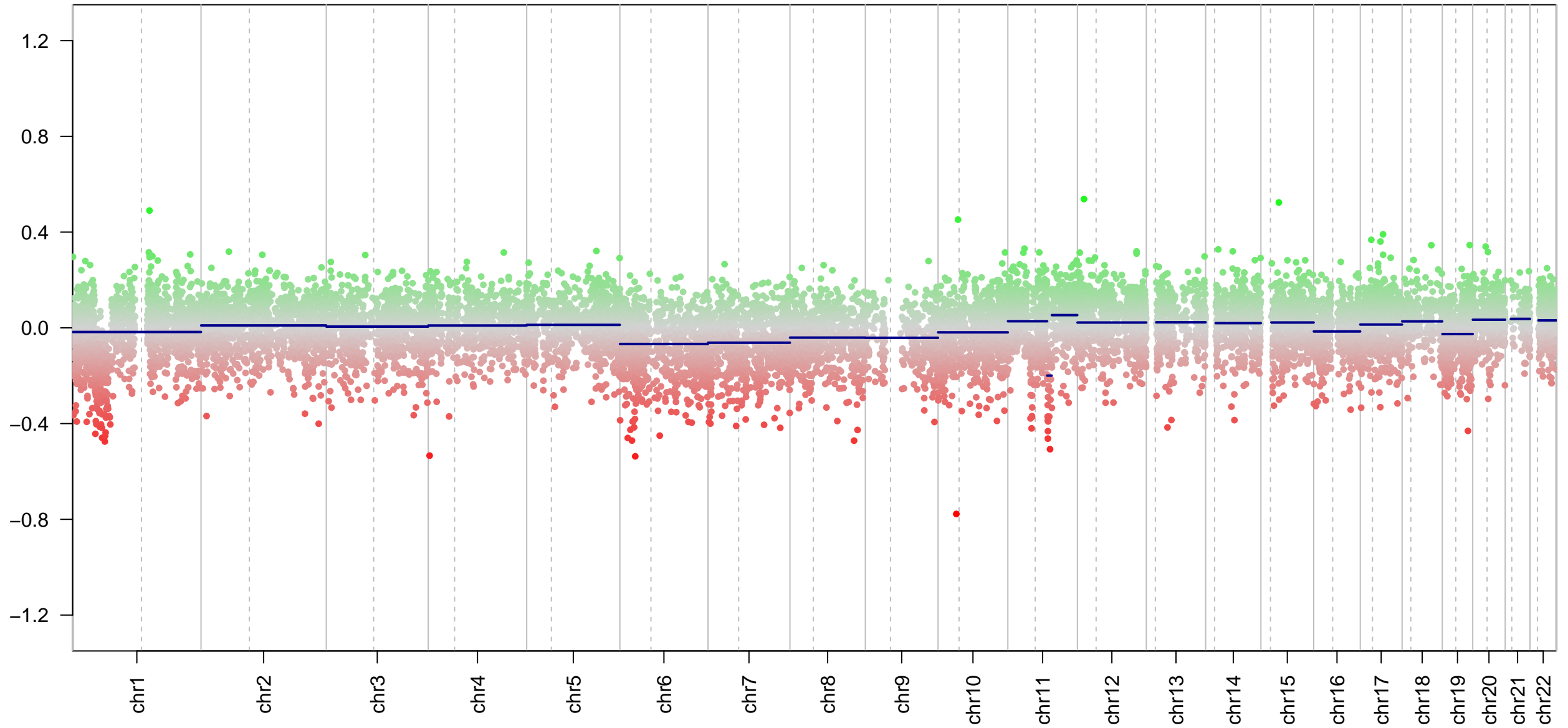

Supplement: Supplementary file 7 — Additional file 7. Copy number variation profile of case #3. [file 40478_2021_1238_MOESM7_ESM.pdf]

Case\_4

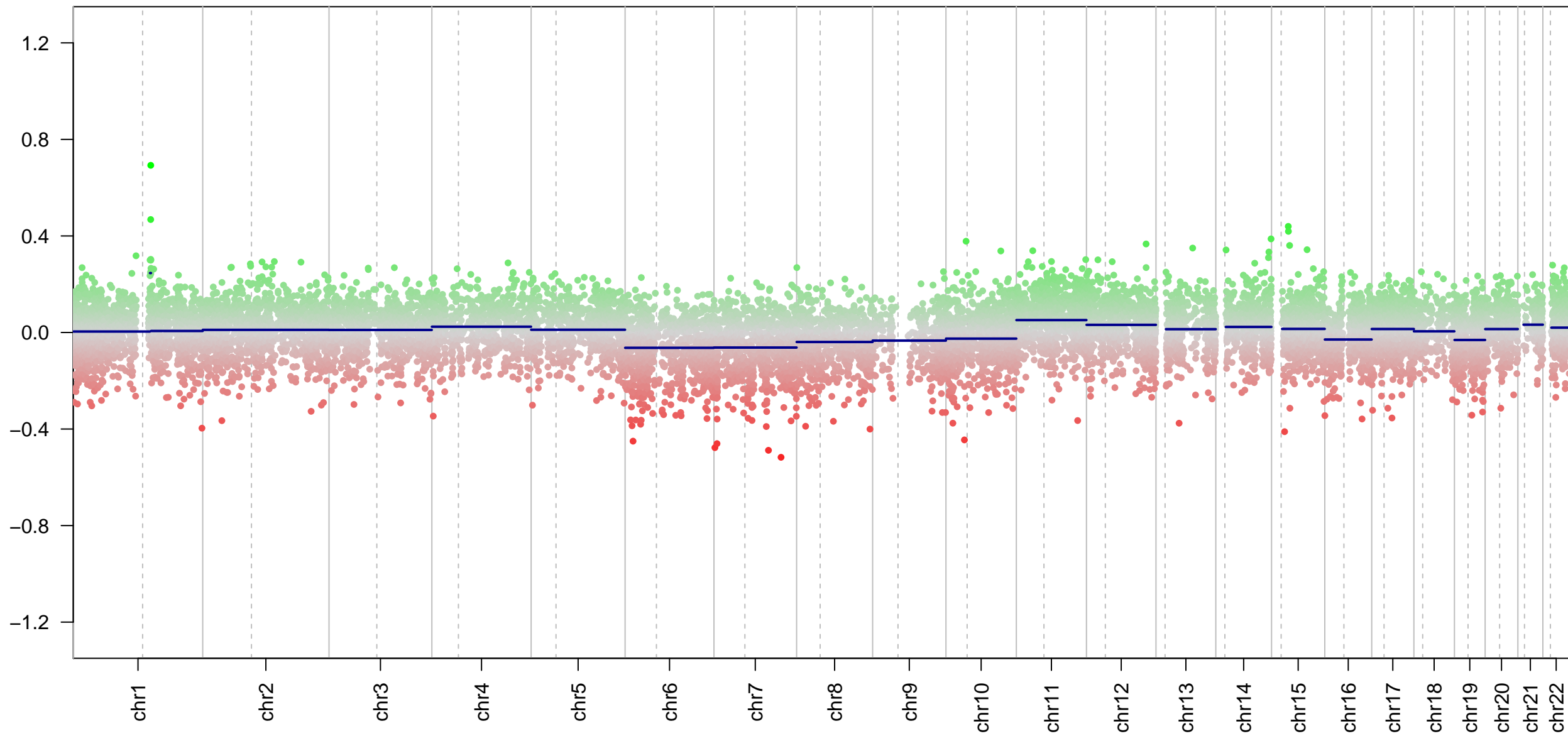

Supplement: Supplementary file 8 — Additional file 8. Copy number variation profile of case #4. [file 40478_2021_1238_MOESM8_ESM.pdf]

Case\_5

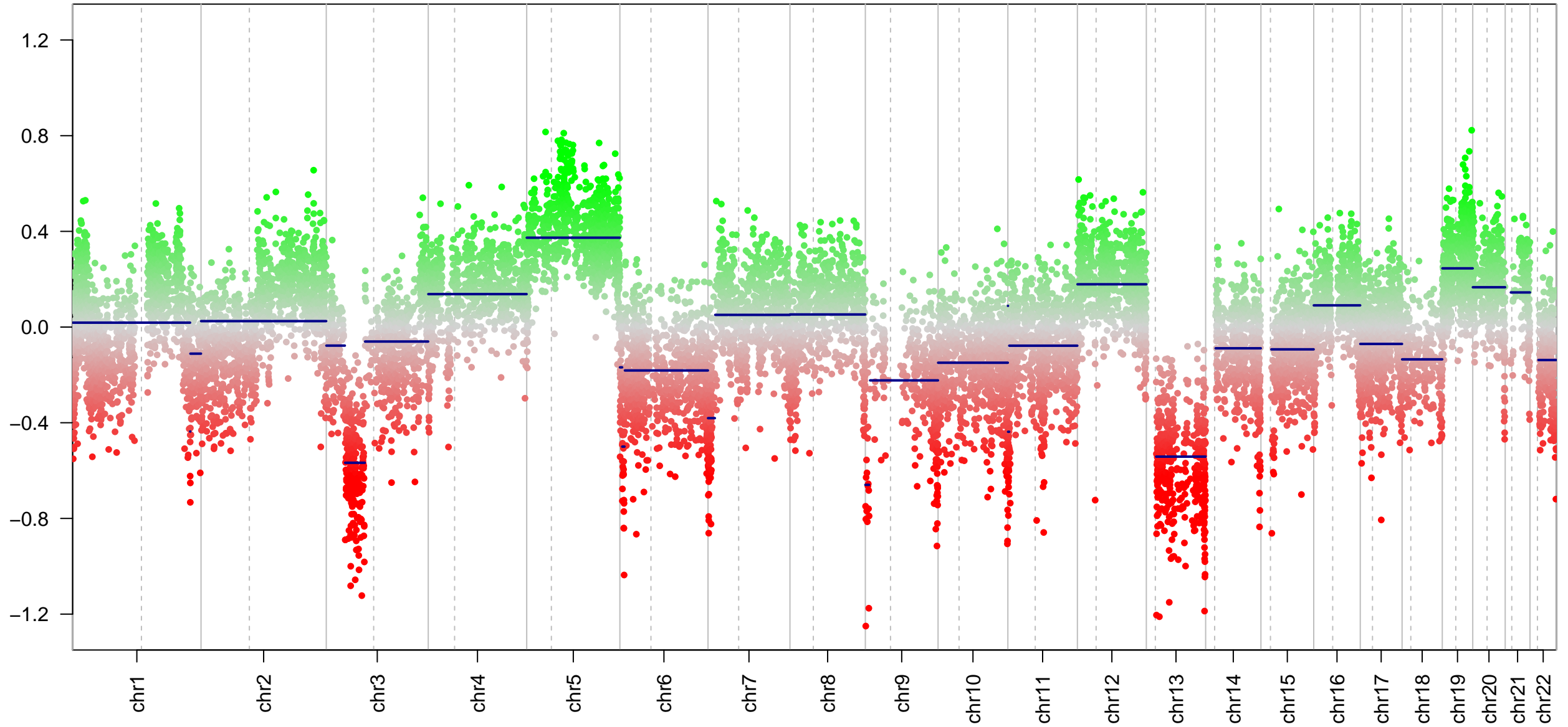

Supplement: Supplementary file 9 — Additional file 9. Copy number variation profile of case #5. [file 40478_2021_1238_MOESM9_ESM.pdf]

Case\_6

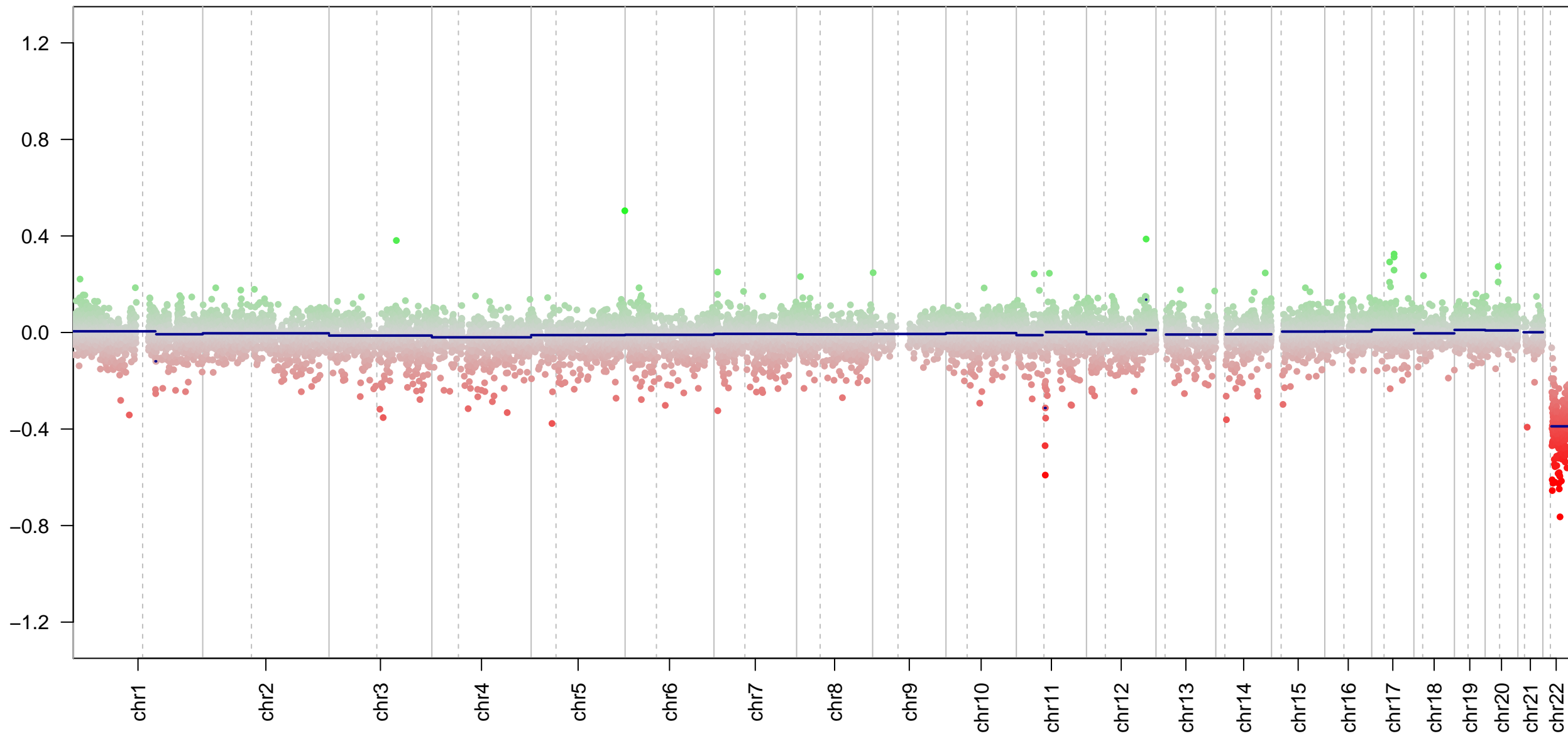

Supplement: Supplementary file 10 — Additional file 10. Copy number variation profile of case #6. [file 40478_2021_1238_MOESM10_ESM.pdf]

Case\_7

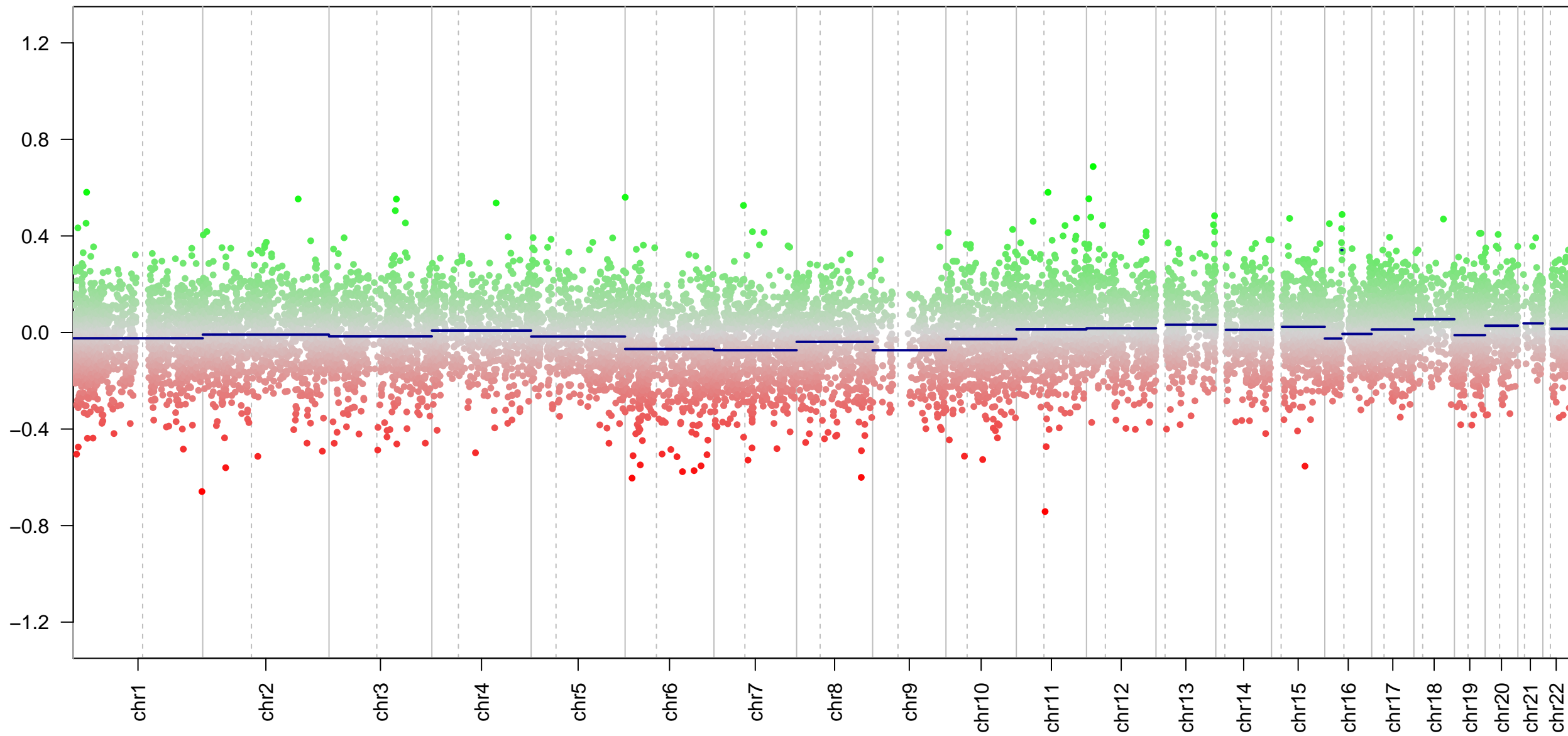

Supplement: Supplementary file 11 — Additional file 11. Copy number variation profile of case #7. [file 40478_2021_1238_MOESM11_ESM.pdf]

Case\_8

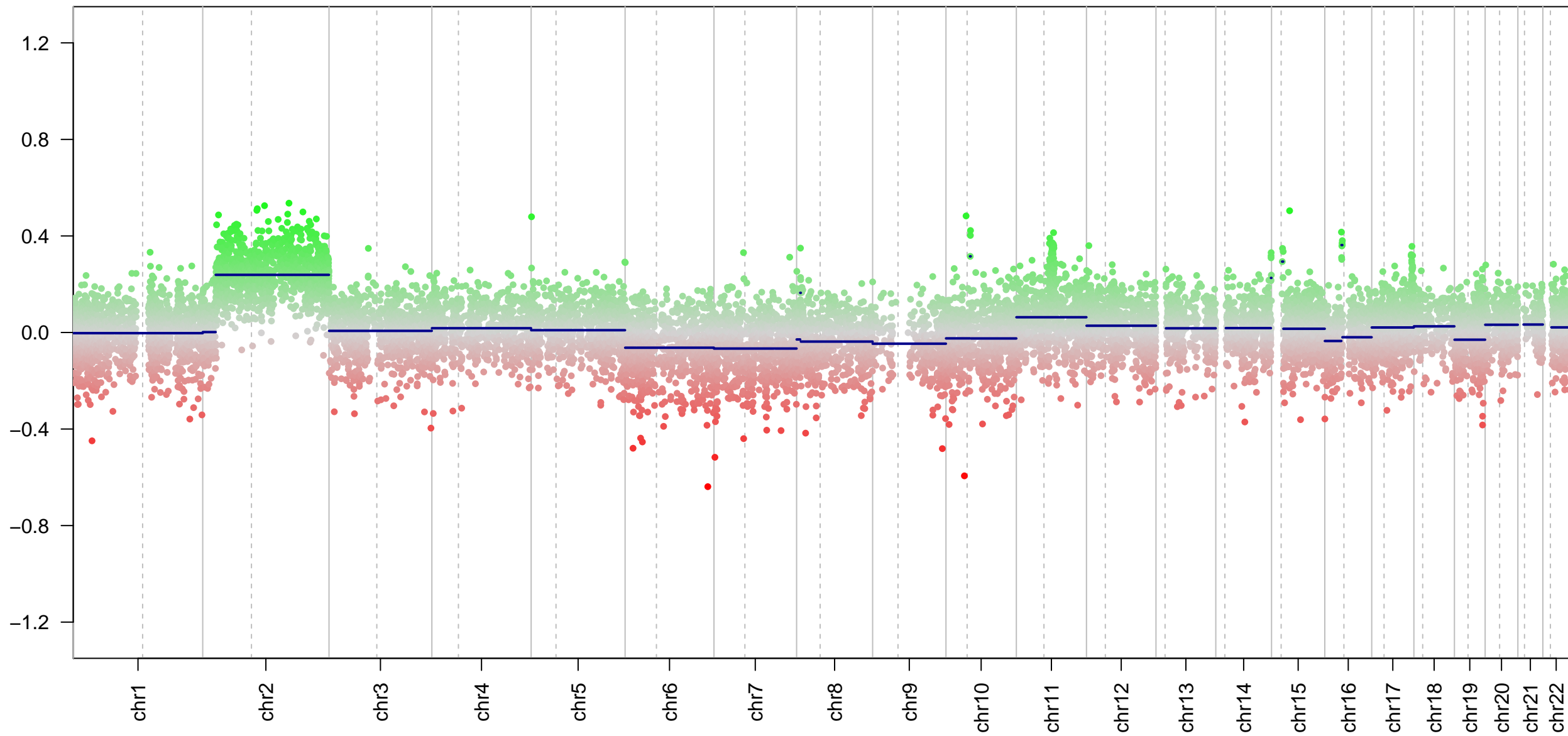

Supplement: Supplementary file 12 — Additional file 12. Copy number variation profile of case #8. [file 40478_2021_1238_MOESM12_ESM.pdf]

Case\_9

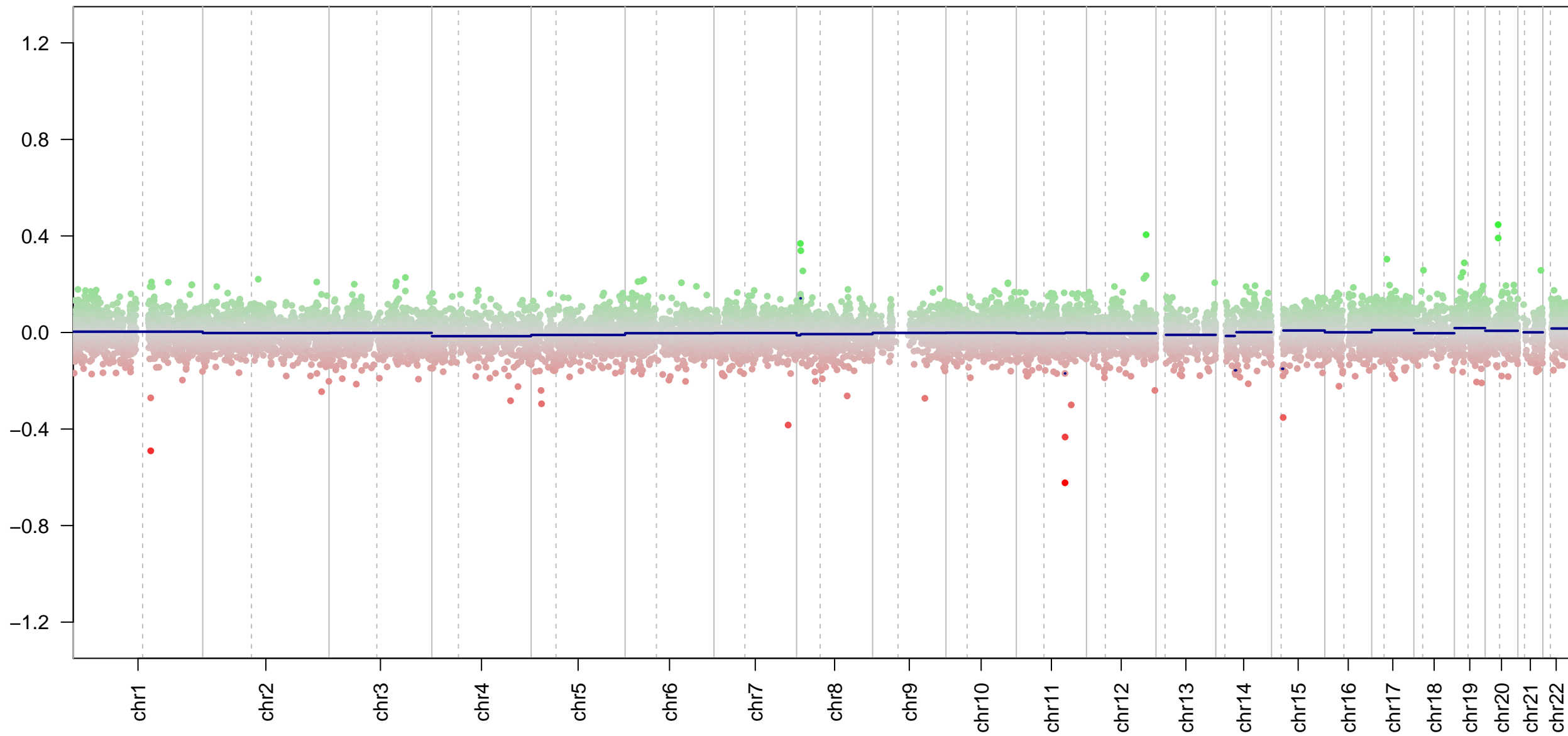

Supplement: Supplementary file 13 — Additional file 13. Copy number variation profile of case #9. [file 40478_2021_1238_MOESM13_ESM.pdf]

Case\_10

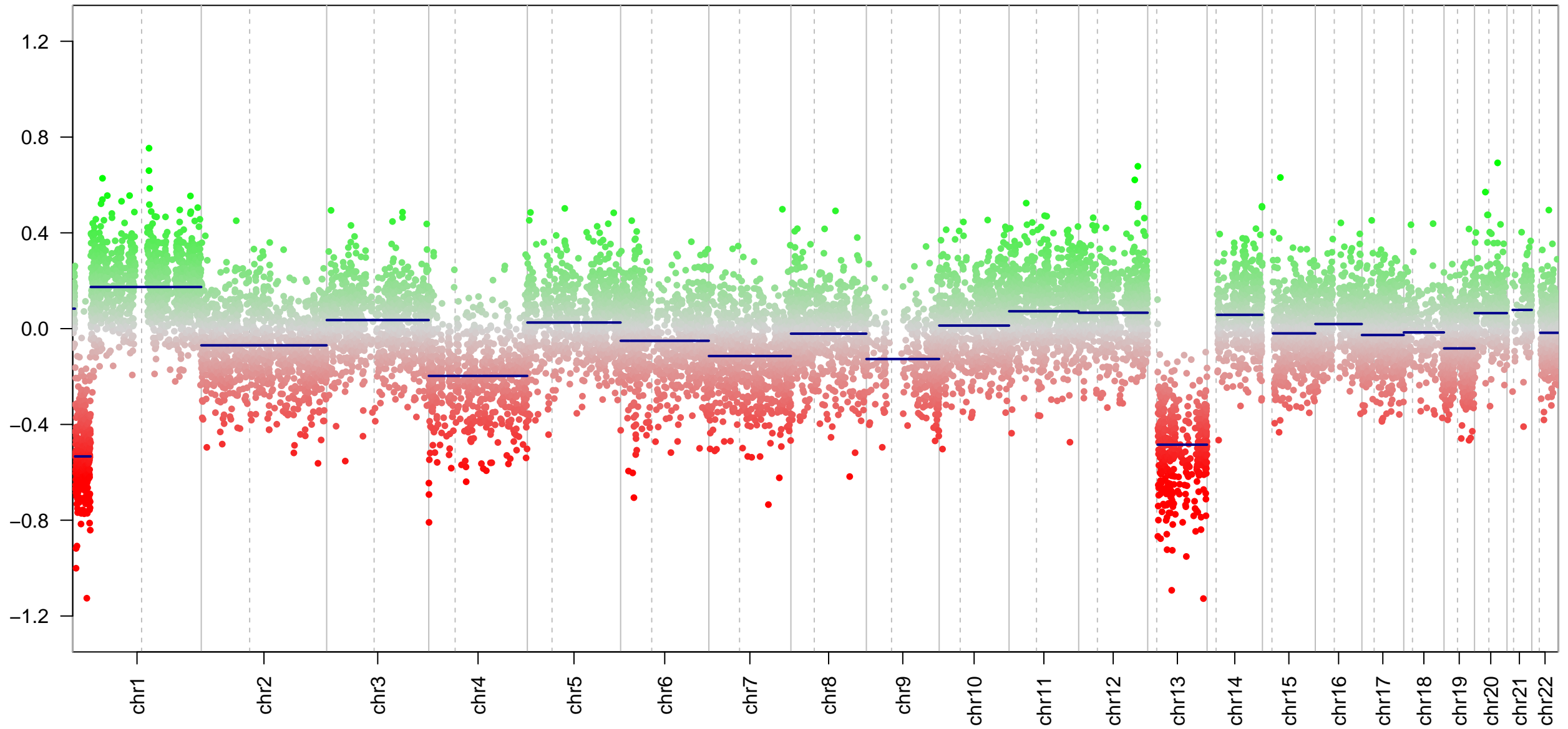

Supplement: Supplementary file 14 — Additional file 14. Copy number variation profile of case #10. [file 40478_2021_1238_MOESM14_ESM.pdf]

Case\_11

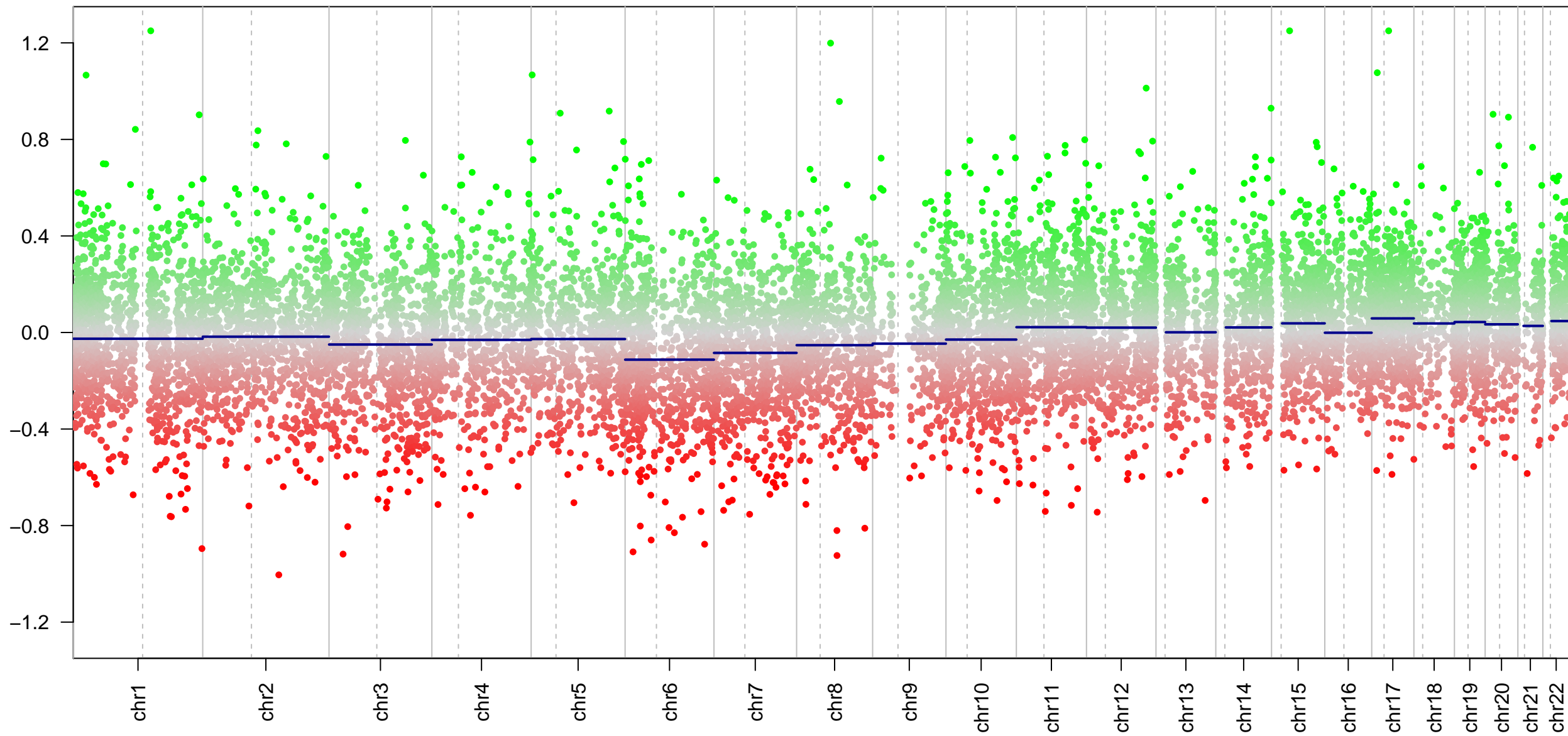

Supplement: Supplementary file 15 — Additional file 15. Copy number variation profile of case #11. [file 40478_2021_1238_MOESM15_ESM.pdf]

Case\_12

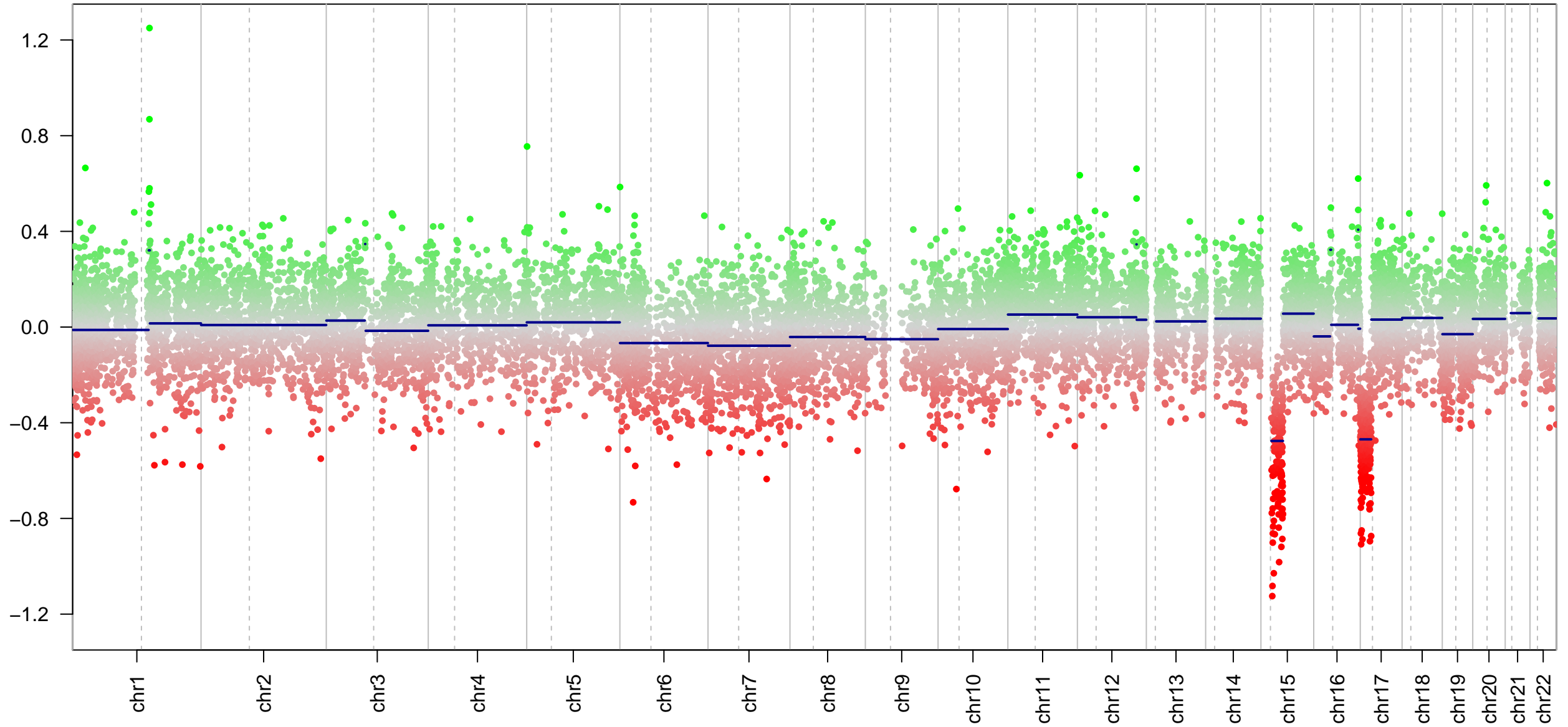

Supplement: Supplementary file 16 — Additional file 16. Copy number variation profile of case #12. [file 40478_2021_1238_MOESM16_ESM.pdf]

Case\_13

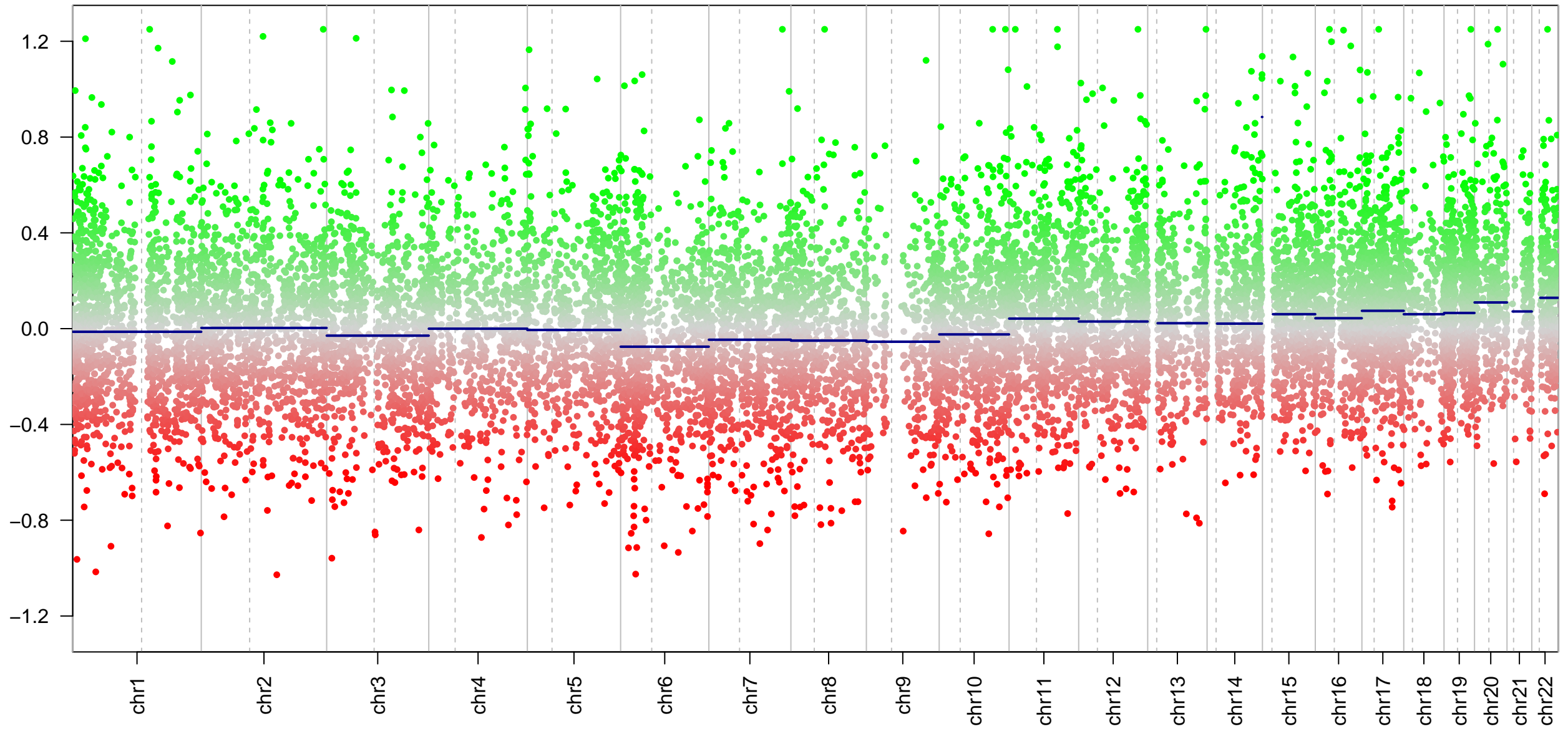

Supplement: Supplementary file 17 — Additional file 17. Copy number variation profile of case #13. [file 40478_2021_1238_MOESM17_ESM.pdf]
